# Molecular and Functional Analyses of a Maize Autoactive NB-LRR Protein Identify Precise Structural Requirements for Activity

**DOI:** 10.1371/journal.ppat.1004674

**Published:** 2015-02-26

**Authors:** Guan-Feng Wang, Jiabing Ji, Farid EI-Kasmi, Jeffery L. Dangl, Guri Johal, Peter J. Balint-Kurti

**Affiliations:** 1 Department of Plant Pathology, North Carolina State University, Raleigh, North Carolina, United States of America; 2 Botany and Plant Pathology, Purdue University, West Lafayette, Indiana, United States of America; 3 Department of Biology, University of North Carolina, Chapel Hill, Chapel Hill, North Carolina, United States of America; 4 Department of Biology and Howard Hughes Medical Institute, Curriculum in Genetics and Molecular Biology, Department of Microbiology and Immunology, University of North Carolina, Chapel Hill, Chapel Hill, North Carolina, United States of America; 5 USDA-ARS Plant Science Research Unit, Raleigh, North Carolina, United States of America; Ohio State University, UNITED STATES

## Abstract

Plant disease resistance is often mediated by nucleotide binding-leucine rich repeat (NLR) proteins which remain auto-inhibited until recognition of specific pathogen-derived molecules causes their activation, triggering a rapid, localized cell death called a hypersensitive response (HR). Three domains are recognized in one of the major classes of NLR proteins: a coiled-coil (CC), a nucleotide binding (NB-ARC) and a leucine rich repeat (LRR) domains. The maize NLR gene *Rp1-D21* derives from an intergenic recombination event between two NLR genes, *Rp1-D* and *Rp1-dp2* and confers an autoactive HR. We report systematic structural and functional analyses of Rp1 proteins in maize and *N*. *benthamiana* to characterize the molecular mechanism of NLR activation/auto-inhibition. We derive a model comprising the following three main features: Rp1 proteins appear to self-associate to become competent for activity. The CC domain is signaling-competent and is sufficient to induce HR. This can be suppressed by the NB-ARC domain through direct interaction. In autoactive proteins, the interaction of the LRR domain with the NB-ARC domain causes de-repression and thus disrupts the inhibition of HR. Further, we identify specific amino acids and combinations thereof that are important for the auto-inhibition/activity of Rp1 proteins. We also provide evidence for the function of MHD2, a previously uncharacterized, though widely conserved NLR motif. This work reports several novel insights into the precise structural requirement for NLR function and informs efforts towards utilizing these proteins for engineering disease resistance.

## Author Summary

The plant hypersensitive defense response (HR) is a rapid, localized cell death, usually occurring upon the recognition of specific pathogen-encoded molecules and consequent activation of nucleotide binding-leucine rich repeat (NLR) proteins. Rp1-D21, a naturally-occurring mutant caused by the recombination of two NLR genes, confers a ‘lesion mimic’, HR-like phenotype in the absence of pathogen infection and provides a powerful tool to investigate the molecular mechanisms of NLR regulation. Here we report the results of a genetic screen in maize that identified novel mutations abrogating Rp1-D21-induced HR. To characterize the function of Rp1-D21, we transiently expressed Rp1-D21 and various derivatives in *Nicotiana benthamiana* to observe the resulting levels of HR. We furthermore examined the protein-protein interactions between and within different Rp1-D21 derivatives. We report novel insights into the precise structural requirements for NLR function and determine the function of a previously undefined motif. These insights enable a better understanding of how NLRs regulate the switch between the resting and the active states.

## Introduction

One of the main plant disease resistance mechanisms is mediated by dominant resistance (*R*) genes with a major effect [1]. Many *R* genes have been cloned from diverse plant species, most of which encode nucleotide binding leucine-rich-repeat (NB-LRR or NLR) proteins [2,3]. Based on the secondary structure of their N-termini, NLRs can largely be subdivided into two classes: one containing a Toll-interleukin 1 receptor (TIR) domain (TIR-NB-LRR, TNL hereafter), and the other a putative coiled-coil (CC) domain (CC-NB-LRR, CNL hereafter). Both TNL and CNL proteins have been identified in dicots, while only CNL proteins have been found in monocots [4].

Each NLR is capable of directly or indirectly recognizing the presence of at least one effector protein, usually produced by a subset of isolates of a single pathogen species [5]. Recognition generally leads to activation of the NLR protein and initiation of signal transduction, resulting in the hypersensitive response (HR), which is often accompanied by the induction of a rapid localized cell death at the point of pathogen penetration [6]. HR can contribute to the halting of pathogen growth. When the corresponding effector is not present, wild-type NLRs are held in an inactive state, largely through self-inhibitory intra-molecular interactions, which may also be facilitated or reinforced by additional host proteins [5,7,8].

In general, plant NLR proteins contain three separable structural domains, an N-terminal CC or TIR domain, a C-terminal LRR domain and a central NB-ARC (ARC: APAF1, *R* gene products and CED-4) domain. The ARC subdomain in plant NLRs is further divided into two separated structural units: ARC1 and ARC2 [9,10]. In the NB-ARC domain, several conserved motifs are recognized; in linear order: P-loop/Kinase-1a/Walker A (henceforce called the P-loop), RNBS (Resistance Nucleotide Binding Site)-A, Kinase-2/Walker B, RNBS-B, RNBS-C, GLPL, RNBS-D, MHD [11]. Among the different motifs, the P-loop and MHD motifs are known to be very important for NLR function.

In their inactive form, NLRs likely have ADP bound to the NB-ARC domain. Activation of NLRs involves the exchange of ADP for ATP through a structural change that is thought to result in an open structure of the proteins [12,13]. The P-loop motif within the NB-ARC domain is thought to be involved in binding ATP/ADP [13]. P-loop mutations in the NLR resistance genes *I-2* from tomato, *M* from flax and *RPM1* from Arabidopsis impair ATP binding and ability to confer HR-based resistance [14,15,16]. Mutations in the conserved MHD-motif lead to autoactivation of NLRs, which is thought to be due to weakened ADP-binding and resulting structural change into an open conformation, favoring nucleotide exchange [15,17].

Autoactivate NLRs, i.e. NLRs that can be activated without the need for a recognition event, can be generated *in vivo* or experimentally via recombination between different NLRs, or through specific point mutations. One example of an *in vivo* recombination event leading to an autoactive NLR is from the maize *Rp1* locus. *Rp1* is a complex locus that carries multiple NLR paralogs. The number of these paralogs varies widely between haplotypes, some carrying more than 50 [18]. The *Rp1-D* haplotype consists of 9 NLR paralogs; *Rp1-dp1* to *Rp1-dp8* which have no known function, and *Rp1-D* itself, which confers resistance against the biotrophic fungal pathogen *Puccinia sorghi*, the causal agent of maize common rust [19,20]. These *Rp1* paralogs are more than 90% identical in nucleotide sequence, allowing them to undergo unequal crossing over and occasional intragenic recombination. An intragenic recombination between paralogs *Rp1-D* and *Rp1-dp2* produced the chimeric gene *Rp1-D21*, which was identified on the basis of its ‘lesion mimic’ phenotype in the absence of pathogen infection [20,21].

We previously demonstrated that many of the hallmarks of pathogen-induced immune response, such as H_2_O_2_ accumulation, increased expression of the defense-related genes *PR1*, *PRms* and *WIP1*, are associated with *Rp1-D21*-mediated lesion phenotype in maize. We also demonstrated that the *Rp1-D21* lesion phenotype is genetic background-, temperature- and light-dependent [22,23]. Recently, we deployed *Rp1-D21* as a tool in a novel enhancer/suppressor screen to identify natural modifiers of the HR [23,24,25,26].

Different NLR *R*-genes appear to have a variety of different structural requirements for proper activation and functioning [27,28,29,30,31,32]. The autoactive nature of Rp1-D21 and the fact that its ‘parental’ proteins, Rp1-D and Rp1-dp2, are not autoactive makes it a very useful tool to explore the molecular requirements for NLR regulation, specifically the switch between inactive and active states.

Here we report the characterization of the activity of Rp1 proteins in maize and in *Nicotiana benthamiana*, a model system widely used for characterization of *R*-gene mediated HR for both dicot and monocot NLRs [27,29,33,34]. Using a combination of genetic, molecular biological, biochemical and computational techniques we derive a model for Rp1 activity which provides a better understanding of how NLRs regulate the switch between the resting and the activation states.

## Results

### Characterization of *Rp1-D21* sequence

**Fig 1:**
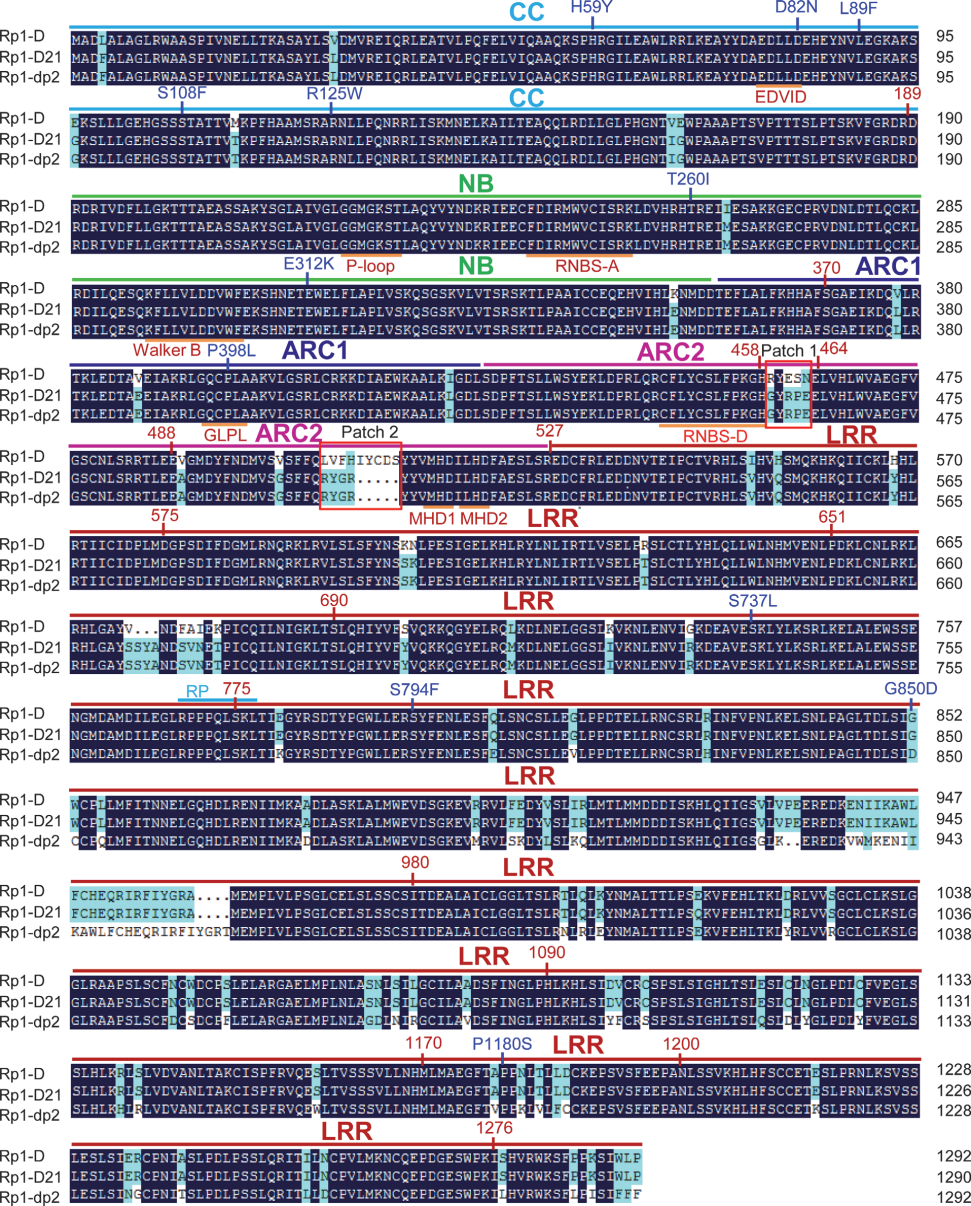
Sequence alignment of Rp1-D, Rp1-dp2 and Rp1-D21. The CC (coiled-coil), NB-ARC (nucleotide binding), ARC1 (APAF1, *R* gene products and CED-4), ARC2 and LRR (leucine-rich-repeat) domains were indicated by bars with cyan, green, dark blue, pink and red colors, respectively. The conserved motifs (EDVID, P-loop, GLPL, RNBS-D, MHD1 and MHD2) were indicated by orange bars and labeled below the sequences. The recombination point of Rp1-D21 was labeled by cyan bar. Patches 1 and 2 are the major difference regions between Rp1-D and Rp1-dp2 in the ARC2 domain. The positions of the mutations in the intragenic Rp1-D21 suppressor from maize were labeled by blue color. The landmark positions of recombination points in the constructs listed in Fig. 6 were labeled by numbers with red color. The black and light blue shaded regions represent 100% and above 50% similarity of the amino acids, respectively.

*Rp1-D21* is derived from the recombination of two NLR paralogs at the *Rp1* locus, *Rp1-D*, and *Rp1-dp2* that are 90% identical at the amino acid level [19,20,21,35]. Sequencing of *Rp1-D21* showed that it encodes a protein of 1290 amino acids (AAs) and is a typical CNL with an N-terminal coiled-coil (CC) domain (AAs 1–189), an NB-ARC domain (AAs 190–527) and an LRR domain at the C-terminus (AAs 528–1290). The N-terminus of *Rp1-D21* derives from *Rp1-dp2* (up to AA 770–778), with the remainder deriving from *Rp1-D* (the precise breakpoint is impossible to define since nucleotides 2310–2333, corresponding to AAs 771–777 are identical in the two progenitor genes, Fig. 1). Thus, Rp1-D21 is comprised of the CC, NB-ARC and the N-terminus of the LRR domain from Rp1-dp2 and the C-terminus of the LRR domain from Rp1-D. A conserved EDVID motif (EDLLD in Rp1-D21, Fig. 1) can be identified in the CC domain of Rp1-D21. All of the important motifs present in the NB-ARC domain of typical NLR proteins can be found in Rp1 proteins (Fig. 1; [11]).

### A targeted EMS-mutagenesis screen identifies intragenic suppressor mutations in *Rp1-D21*

**Fig 2:**
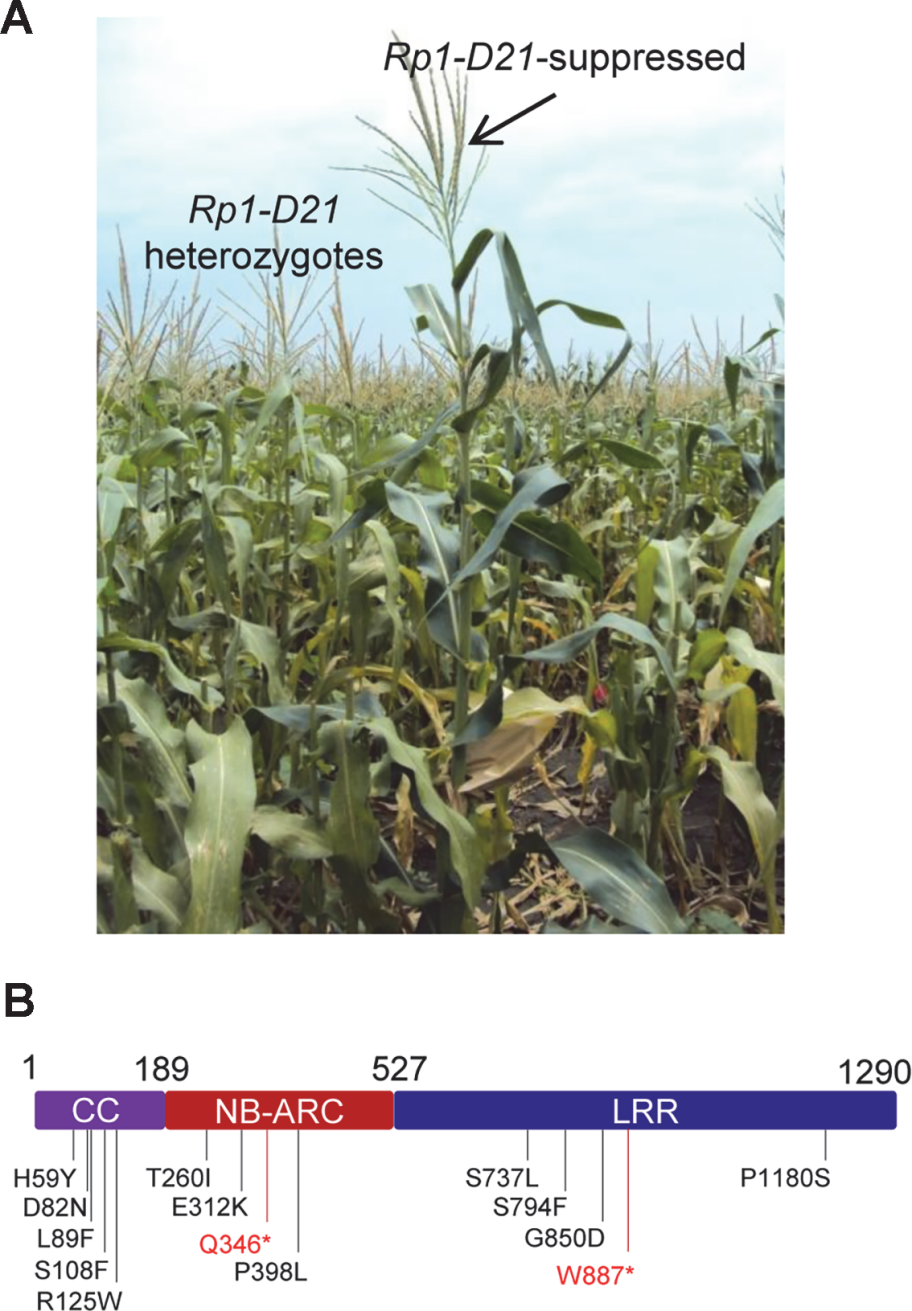
EMS mutagenesis screening for mutants that lost the hypersensitive response (HR) induced by *Rp1-D21*. (A) The growth phenotype of *Rp1-D21* heterozygotes in the field compared to the *Rp1-D21* mutant in which the HR phenotype is suppressed (arrow). (B) Twelve missense (in black) and two nonsense (in red) point mutations that abolished the HR phenotype of *Rp1-D21* were identified in different domains (CC, NB-ARC and LRR) of Rp1-D21.

We performed a targeted ethyl methanesulfonate (EMS) mutagenesis screen in a maize line harboring *Rp1-D21* to identify mutations that lost the autoactive HR phenotype conferred by *Rp1-D21*. Putative suppressor mutants were easily identified due to their robust growth compared to the stunted, lesion-mimic siblings heterozygous for *Rp1-D21* (Fig. 2A). From about 23,000 EMS-mutated M1 plants, 12 missense and 2 nonsense intragenic mutants were identified (Fig. 2B; Table 1). Among the 12 missense mutants, five had mutations in the CC domain, three in the NB-ARC domain and four in the LRR domain (Fig. 2B). Notably, one mutant (nucleotide G244A, thus D82N in amino acid) was in the conserved EDVID motif of the CC domain, and one (C1193T, P398L) in the conserved GLPL motif of the NB-ARC domain (Table 1; Figs. 1 and 2B). The mutants T260I and E312K were adjacent to the conserved RNBS-A and Walker B motifs, respectively (Table 1; Figs. 1 and 2B).

**Table 1:** Mutations that abrogated *Rp1-D21*-induced HR identified in an EMS mutagenesis screen in maize.

Domain	Nucleotide mutation	Amino acid change	HR rating of full lengh in *N*. *benthamiana*	HR rating of the CC domain in *N*. *benthamiana*	Self-association of the CC domain	Conserved motif
Rp1-D21			4	4	+ ^b^	
CC	C175T	H59Y	4	4	+	
CC	G244A	D82N	0	5	+/-	In EDVID
CC	C265T	L89F	2	2	+	
CC	C323T	S108F	3	2	+	
CC	C373T	R125W	0	0	+	
NB-ARC	C779T	T260I	1			Next to RNBS-A
NB-ARC	G934A	E312K	2			Next to walker B
NB-ARC	C1036T	Q346*^a^				
NB-ARC	C1193T	P398L	0.5			In GLPL motif
LRR	C2210T	S737L	2			
LRR	C2381T	S794F	0			
LRR	G2549A	G850D	4			
LRR	G2660A	W887*^a^				
LRR	C3538T	P1180S	2			

^a^ *indicates stop codon.

^b^ +: strong self-association;

+/-: weak self-association.

### The HR phenotype conferred by Rp1-D21 in maize can be recapitulated by transient expression in *N*. *benthamiana*

**Fig 3:**
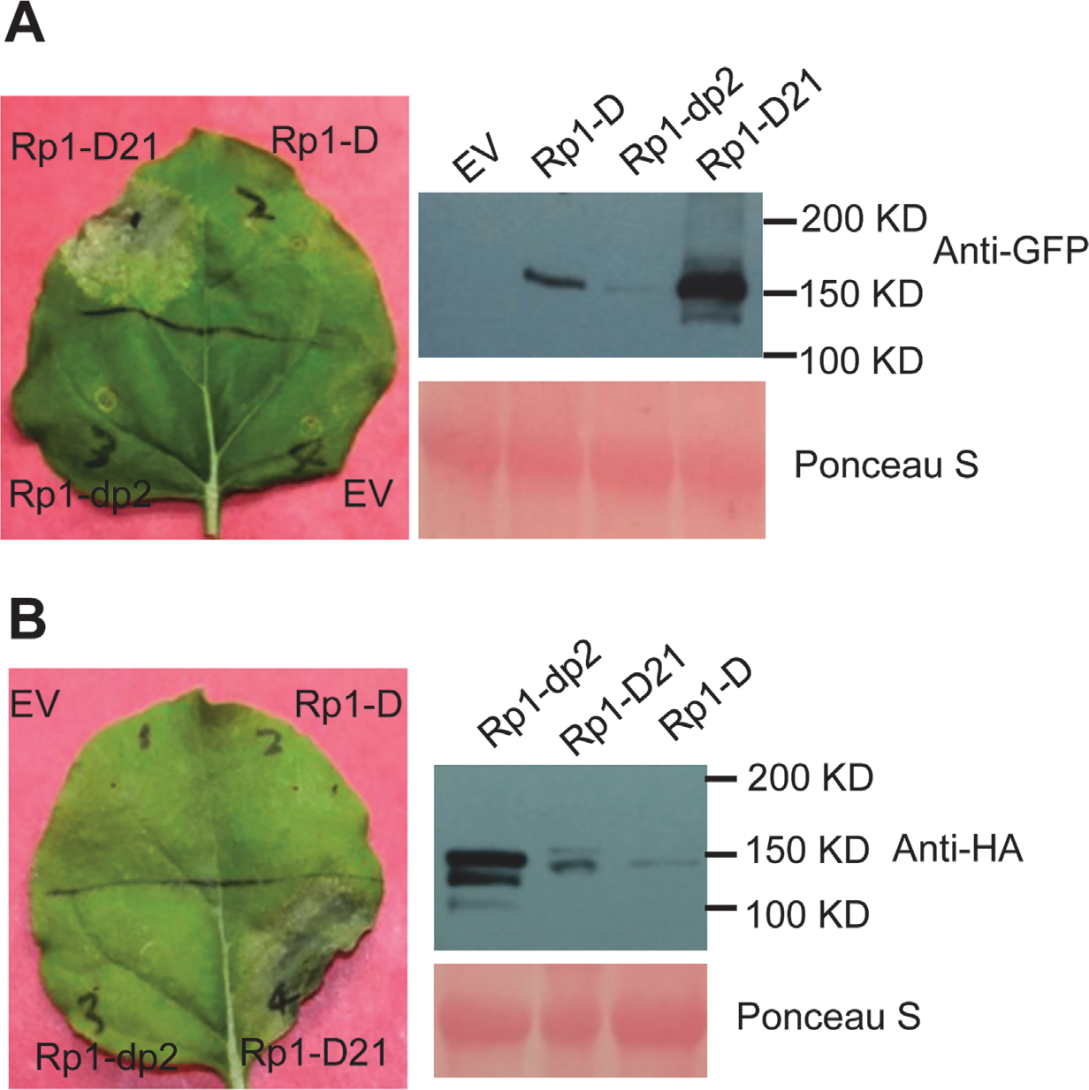
Rp1-D21 triggers a hypersensitive response phenotype when transiently expressed in *N*. *benthamiana*. (A) Rp1-D21, Rp1-D and Rp1-dp2 proteins fused with a C-terminal EGFP tag were agro-infiltrated into *N*. *benthamiana*, with an empty vector (EV) as a negative control. A representative leaf was photographed at 3 days post infiltration (dpi, left). Total protein was extracted from agro-infiltrated leaves at 30 hours post infiltration (hpi), and anti-GFP antibody was used to detect the expression of the fused proteins (right). The sizes of the proteins were labeled on the right. Equal loading of protein samples was shown by Ponceau-S staining of the Rubisco subunit (below, right). (B) Rp1-D21, Rp1-D and Rp1-dp2 proteins fused with a C-terminal 3×HA tag were agro-infiltrated into *N*. *benthamiana*, with empty vector (EV) as a negative control. A representative leaf was photographed at 3 dpi (left). Total protein was extracted from agro-infiltrated leaves at 30 hpi, and anti-HA antibody was used to detect the expression of the fused proteins. Equal loading of protein samples was shown by Ponceau-S staining of Rubisco subunit (below, right). These experiments were repeated three times with the same results.

We used Agrobacterium-mediated transient expression in *N*. *benthamiana* to investigate the structure/function of Rp1-D21. Rp1-D, Rp1-dp2 and Rp1-D21 were either fused to the N-terminus of enhanced green fluorescent protein (EGFP), a 3×HA (influenza hemagglutinin) tag or not tagged for subsequent functional analysis. When the three fusion proteins were transiently expressed using the cauliflower mosaic virus 35S promoter, a HR phenotype was observed 3 days post-infiltration (dpi) only with Rp1-D21, but not with Rp1-D, Rp1-dp2 or the empty vector (EV) control. The same phenotypes were obtained regardless of the tags used (EGFP, 3×HA or no tag) and regardless of the differing levels of protein accumulation observed for the three proteins (Fig. 3; S1A Fig.).

To further confirm that the phenotype conferred by Rp1-D21 in our *N*. *benthamiana* system conformed to the phenotypes observed in maize, we constructed Rp1-D21 expression vectors carrying the missense Rp1-D21 suppressor mutations identified in maize. When transiently expressed in *N*. *benthamiana*, Rp1-D21 produced a strong HR phenotype, rating 4 based on a 0–5 scale rating with 0 being no cell death or chlorosis at all and 5 being confluent cell death [36]. The HR rating is based on the average results from at least 10 individual leaves. All the mutants we identified as non-functional in maize, except for H59Y and G850D, abolished or greatly reduced the level of Rp1-D21-induced HR (Table 1; S2A Fig.). All constructs conferred high levels of protein expression (S2B Fig.). The concordance between the maize and *N*. *benthamiana* systems is very good and we expect that our conclusions based on the transient expression *N*. *benthamiana* system are generally relevant to the endogenous maize system. Thus, we used the transient expression of Rp1-D21 and its derivatives in *N*. *benthamiana* to further analyze the molecular mechanism of activation/auto-inhibition of Rp1 proteins.

### Rp1 protein activity depends on P-loop and MHD motifs

**Fig 4:**
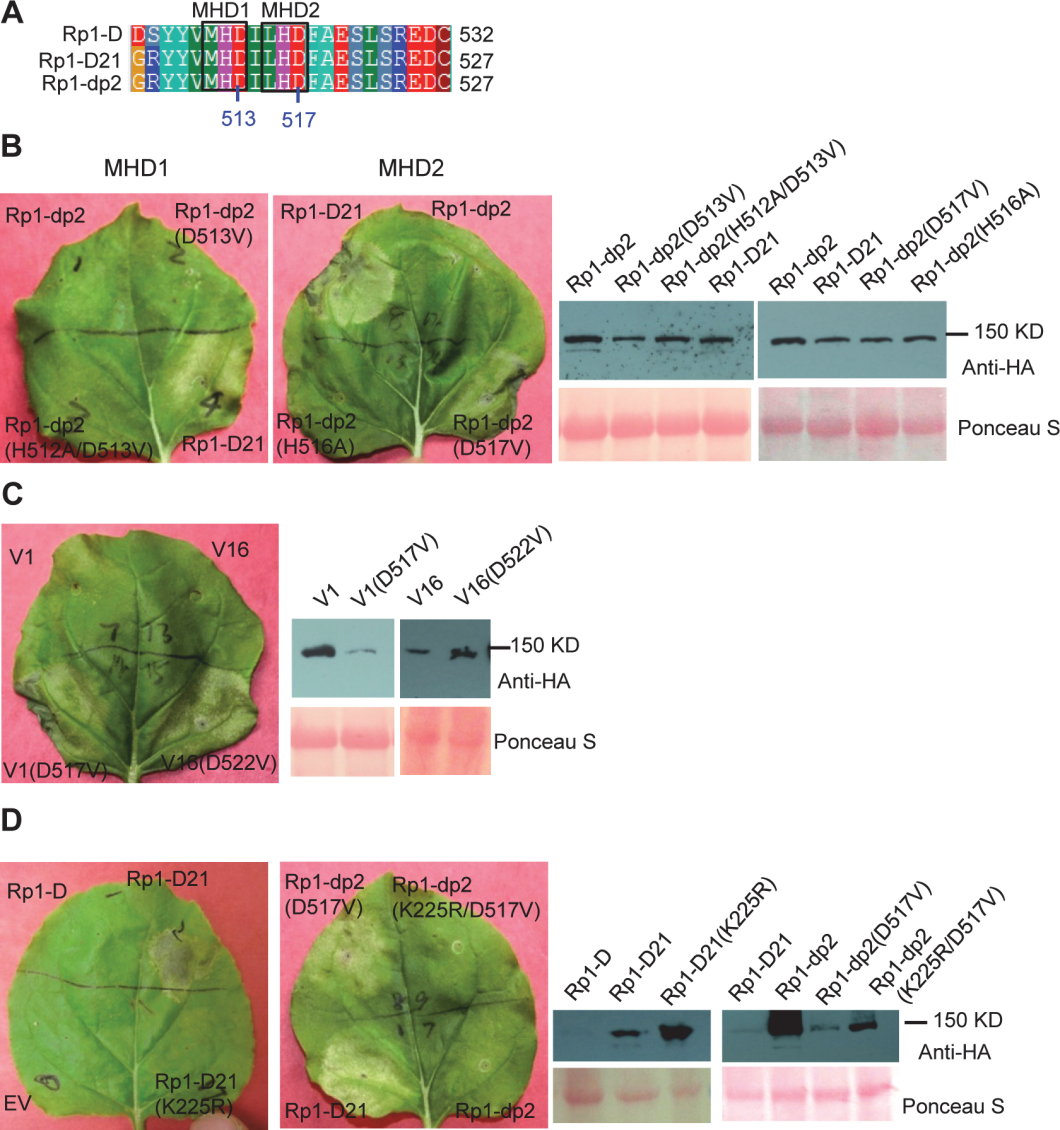
Functional characterization of the MHD2 motif and the P-loop motif. (A) Multiple sequence alignment of the MHD motifs from Rp1-D, Rp1-D21 and Rp1-dp2. Positions of the MHD1 and MHD2 motifs are indicated. The numbers indicated the positions of the aspartate (D) corresponding to Rp1-dp2. (B) The MHD1 and MHD2 point mutations in Rp1-dp2 background were generated and transiently expressed in *N*. *benthamiana*. (C) Investigating the MHD2 point mutations in V1 and V16, two recombinant constructs that were not autoactive as shown in Fig. 6. (D) Point mutations in P-loop motif of Rp1-D21 and Rp1-dp2(D517V) caused the loss of the ability to induce HR. EV (empty vector) as a negative control. The picture of hypersensitive response (HR) phenotype was taken at 3 days post-infiltration and total protein was detected by anti-HA antibody from agro-infiltrated leaves at 30 hours post infiltration. Equal loading of protein samples was shown by Ponceau-S staining of Rubisco subunit. The experiments were repeated three times with the same results.

The MHD motif is a highly conserved region of the NB-ARC domain involved in nucleotide binding [15]. Mutations in the MHD domain confer autoactivity to a number of NLRs. Two MHD motifs, here termed MHD1 and MHD2 (actually LHD in Rp1-D and Rp1-dp2; Fig. 4A), separated by a single amino acid are apparent in Rp1-D, Rp1-dp2 and Rp1-D21, and in a number of other CNLs [37]. To investigate whether mutations in either of the MHD motifs could cause autoactivity in the non-autoactive parental proteins, Rp1-D and Rp1-dp2, we generated the following mutations: Rp1-D(H517A), Rp1-D(D518V), Rp1-dp2(D513V) and Rp1-dp2(H512A/D513V) in the MHD1 motif, and Rp1-D(H521A), Rp1-D(D522V), Rp1-dp2(H516A), Rp1-dp2(D517V) in the MHD2 motif. Transient expression of the Rp1-dp2(D517V) MHD2 mutant conferred an obvious HR, with symptoms appearing at 3.5 days, about 12 h later than that observed with Rp1-D21 (Fig. 4B). HR was not induced by transient expression of any of the other MHD mutants, including the Rp1-D(D522V) MHD2 mutation (S3 Fig.). However, Rp1-D(D522V) expression was not detectable by western blot analysis using anti-HA antibody. Protein expressed from all the other constructs could be detected (Fig. 4B; S3 Fig.). We also generated the MHD2 mutations V1(D517V) and V16(D522V) in V1 and V16, two recombinant constructs that were not autoactive (see below) and found both of them induced HR (Fig. 4C).

The P-loop is a highly conserved motif in NLRs and mutations in this domain often result in loss of function and loss of autoactivity [14,15,27,34,38,39]. P-loop mutations (K225R) introduced into Rp1-D21 and the autoactive Rp1-dp2(D517V) mutant abrogated their ability to confer HR (Fig. 4D). This indicated that the Rp1-D21 autoactivity and presumably also the activity of the Rp1 proteins are P-loop dependent.

### The CC domain is sufficient for inducing HR

**Fig 5:**
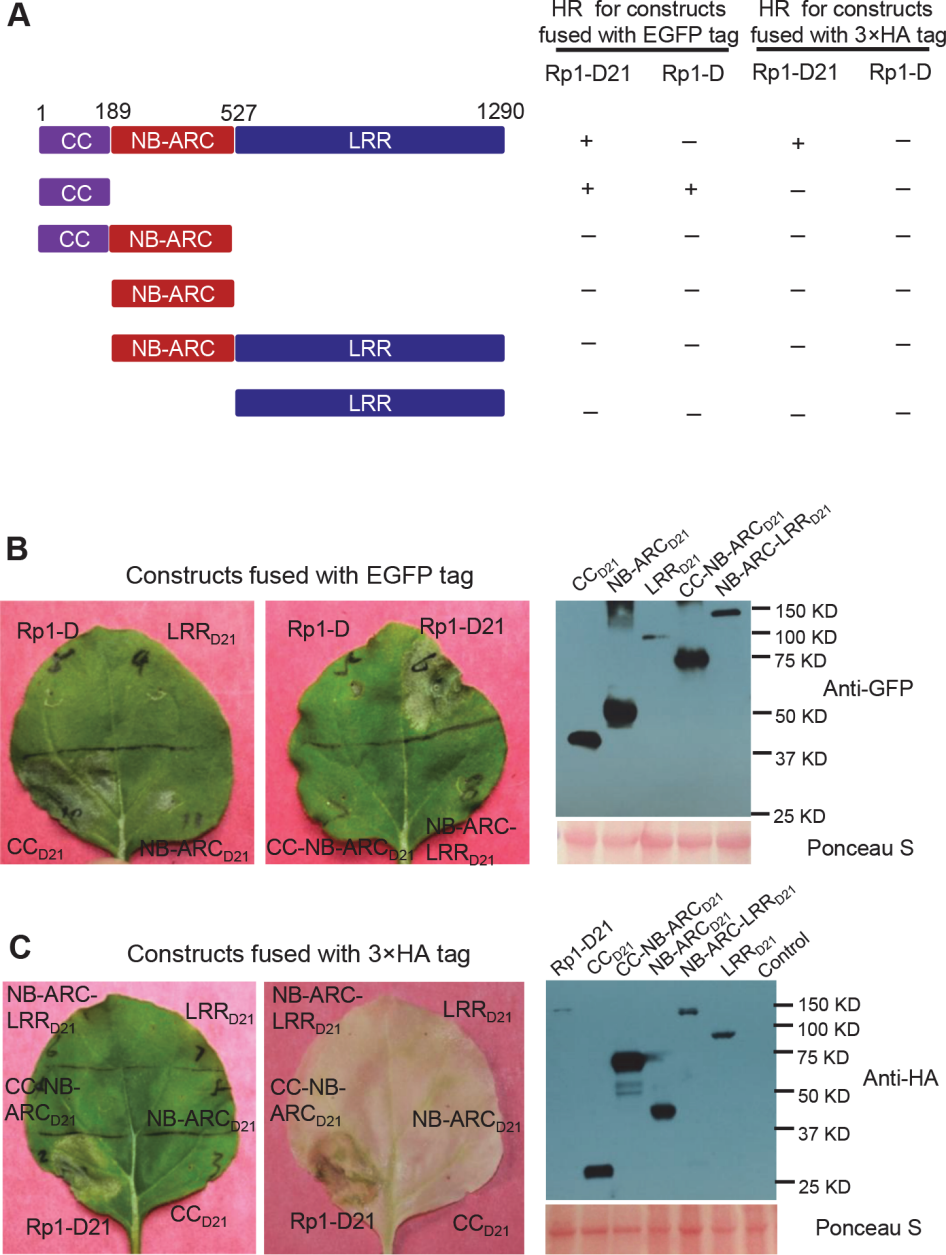
Investigating the functional domains of Rp1-D21 and Rp1-D to trigger a hypersensitive response (HR). (A) Schematic diagram of the Rp1-D21 and Rp1-D domain structure and the derived fragments used for agro-infiltration of *N*. *benthamiana*. The different domains are indicated using different colors: CC (purple); NB-ARC (red); LRR (dark blue). The positions of the amino acids that form the domain boundaries are indicated on the top and the abilities to induce (+) or not induce (-) HR are listed on the right of each construct. (B) The HR phenotype of the constructs fused with a C-terminal EGFP tag and transiently expressed in *N*. *benthamiana*. Representative leaves were photographed at 3 days post infiltration (dpi, left). Total protein was extracted from agro-infiltrated leaves at 30 hours post infiltration (hpi), and anti-GFP antibody was used to detect the expression of fused proteins. Equal loading of protein samples was shown by Ponceau-S staining of Rubisco subunit (right, below). The results shown were from different domains of Rp1-D21, and similar results were observed from different domains of Rp1-D. (C) The phenotype of the constructs fused with a C-terminal 3×HA tag and transiently expressed in *N*. *benthamiana*. A representative leaf was photographed at 3 dpi (left), and the same leaf was cleared by ethanol (middle). Total protein was extracted from agro-infiltrated leaves at 30 hpi, and anti-HA antibody was used to detect the expression of the fused proteins. Equal loading of protein samples was shown by Ponceau-S staining of Rubisco subunit (right). The results shown were from different domains of Rp1-D21, and similar results were observed from different domains of Rp1-D. The experiments were repeated three times with the same results.

To examine which region of Rp1-D21 was required for triggering HR, different domains or domain combinations of Rp1-D21, including CC, CC-NB-ARC, NB-ARC, NB-ARC-LRR and LRR domains (hereafter CC_D21_, CC-NB-ARC_D21_, NB-ARC_D21_, NB-ARC-LRR_D21_ and LRR_D21_; Fig. 5A), were fused to the N-terminus of EGFP. CC_D21_ and NB-ARC_D21_ were derived from (and are therefore identical to) the corresponding domains of Rp1-dp2 while LRR_D21_ is recombinant between the LRRs of Rp1-D and Rp1-dp2. The transient expression of CC_D21_, but of no other Rp1-D21 domains or domain combinations, conferred HR (Fig. 5B). CC_D21_, NB-ARC_D21_ and CC-NB-ARC_D21_ produced higher protein accumulation than NB-ARC-LRR_D21_ and LRR_D21_ (Fig. 5B), excluding the possibility that lack of HR phenotype seen with most of the domain constructs was solely due to low protein accumulation. We also tested the HR phenotype conferred by different Rp1-D domains and found that the CC domain, but no others, induced HR when fused with EGFP (Fig. 5A). Surprisingly, no untagged or HA-tagged domains from either Rp1-D or Rp1-D21 induced HR (Fig. 5; S1B Fig.).

CC_D21_, but not CC-NB-ARC_D21_, induced HR, which suggested that NB-ARC_D21_ can inhibit CC_D21_-induced HR *in cis* (when the two domains were fused in the same molecule). Consistent with this result, no HR was observed when EGFP-tagged CC_D21_ and NB-ARC_D21_ were transiently co-expressed *in trans* (when the two domains were co-expressed as separate molecules) in *N*. *benthamiana* (Table 2). LRR_D21_ did not restore HR when co-expressed with CC_D21_ and NB-ARC_D21_ separately, or with CC-NB-ARC_D21_ *in trans*. However, co-expressing CC_D21_ with NB-ARC-LRR_D21_ retained HR *in trans*, suggesting that LRR_D21_ might interact with NB-ARC_D21_ to regulate CC_D21_–induced HR *in cis* but not *in trans* (Table 2). However, since LRR_D21_ or NB-ARC-LRR_D21_ are expressed at substantially lower levels than CC_D21_ or CC-NB-ARC_D21_ (Figs. 5B and 5C), we cannot exclude the possibility that the concentration of LRR_D21_ or NB-ARC-LRR_D21_ is simply too low to affect the NB-ARC suppression of CC_D21_–induced HR or CC_D21_–induced HR itself when co-expressed *in trans*. In contrast to the *in cis* result of CC-NB-ARC_D_, we found that NB-ARC_D_ did not suppress CC_D_-induced HR *in trans* (Table 2), suggesting NB-ARC_D_ might interact with CC_D_ *in cis* but not *in trans*.

**Table 2:** Summary of the HR phenotype conferred by transient expression in *Nicotiana benthamiana* of specific domains or domain combinations from Rp1-D and Rp1-D21 *in cis* and *in trans* (i.e the two or three listed domains either fused in the same molecule in the order presented here, or expressed as separate molecules, respectively).

Domain combinations	HR in cis	HR in trans
CC_D_	+	N/A
CC-NB-ARC_D_	-	N/A
CC_D21_	+	N/A
CC-NB-ARC_D21_	-	N/A
NB-ARC-LRR_D21_	-	N/A
CC_D_ + NB-ARC_D_	-	+
CC_D21_ + NB-ARC_D21_	-	-
CC_D21_ + NB-ARC_D21_ + LRR_D21_	+	-
CC_D21_ + NB-ARC-LRR_D21_	+	+
CC-NB-ARC_D21_ + LRR_D21_	+	-

All constructs are tagged with EGFP at the C-terminus. +: HR; -: without HR; N/A: not applicable.

To further investigate the inhibition region of NB-ARC in CC-induced HR, we generated a series of deletion constructs from CC-NB-ARC (S4 Fig.). We found that extension including AAs 190–260 from the NB domain was sufficient to suppress CC-induced HR. Consistent with the data, the nonsense EMS mutant (Q346*) suppressed the Rp1-D21 lesion-mimic and stunted growth phenotype in maize (Fig. 2B; Table 1).

The C-terminus of the LRR domain has been demonstrated to be important for NLR activity [37]. We therefore investigated whether C-terminal deletions of the LRR domain from Rp1-D21 affected the HR phenotype. None of the six C-terminal deletion constructs (ranging from 34 to 639 AAs) induced HR after transient expression, even D21-LRR27 that lacked only the last 34 AAs of the acidic tail in C-terminus (S5 Fig.). This is despite the fact that all the constructs conferred high levels of protein expression (S5B Fig.).

### Domain swaps between Rp1-D and Rp1-dp2 delineate precise requirements for self-inhibition

**Fig 6:**
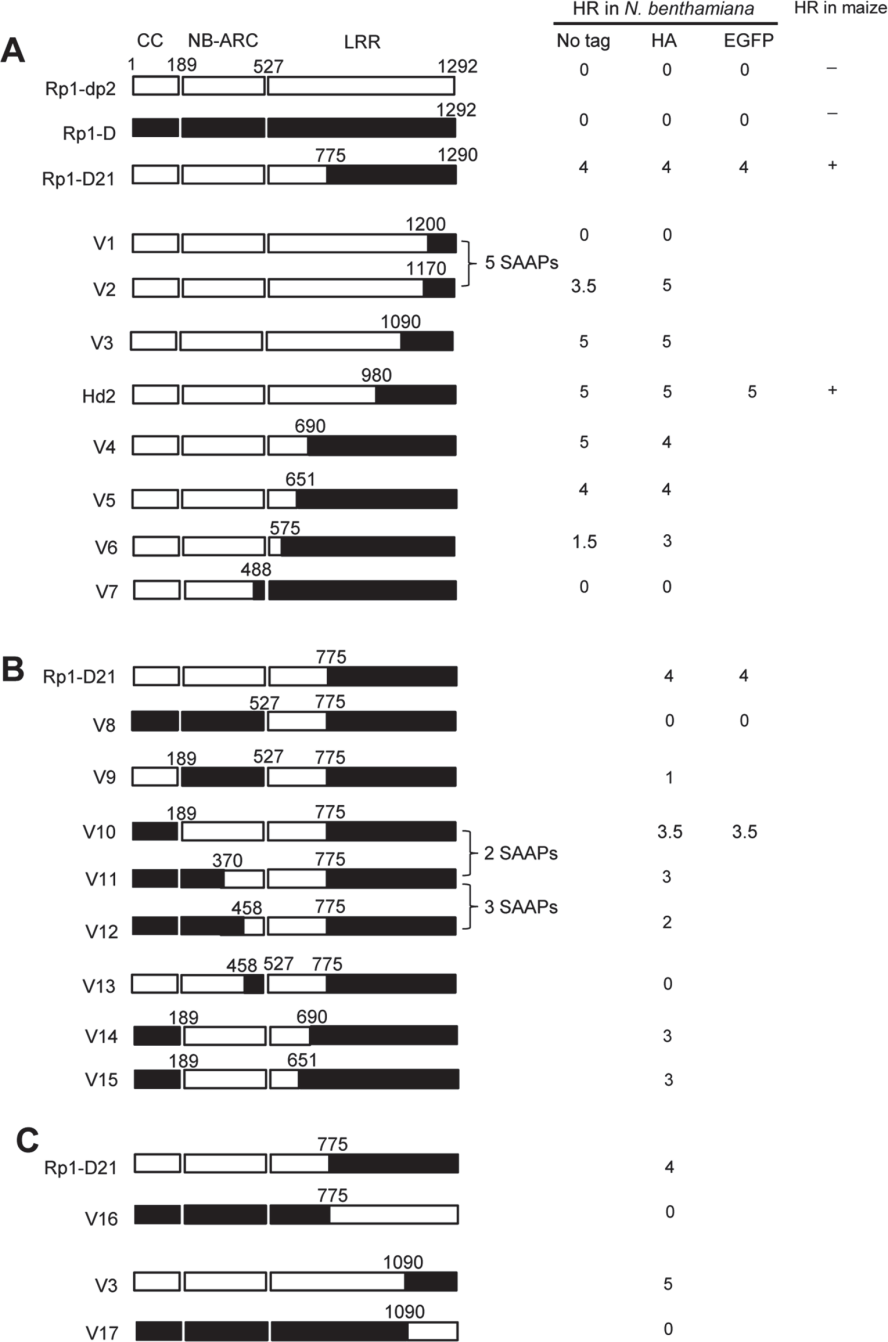
Schematic diagram of domain swap constructs between Rp1-D and Rp1-dp2 used for transient expression in *N*. *benthamiana*. For ease of interpretation, the gene structure has been divided into CC (coiled coil), NB-ARC (nucleotide binding) and LRR (leucine rich repeat). The amino acid position of the recombination site of each construct was indicated above the construct. For each construct, either with no tag, 3×HA tag or EGFP tag, the strength of the hypersensitive response (HR) resulting from transient expression in *N*. *benthamiana* is shown. HR was scored on a 0 (no HR) to 5 (strong HR) scale according to Slootweg et al. (2013). If there was no score recorded, the particular experiment was not performed. Where known, the abilities of constructs to induce (+) or not induce (-) HR in maize were indicated. In some cases the number of single amino acid polymorphisms (SAAP) between constructs is indicated. All experiments were performed three times with similar results. (A) Constructs with the N-terminus from Rp1-D and C-terminus from Rp1-dp2. (B) Constructs designed to delimit the domains of Rp1-D or Rp1-dp2 important for self-inhibition. (C) The reciprocal constructs: Rp1-D21 with V16, and V3 with V17.

To delineate the functional regions that cause Rp1-D21 to be autoactive and keep its progenitor proteins auto-inhibited, we generated a series of chimeric constructs recombinant at different positions between Rp1-D and Rp1-dp2 (Fig. 6). These proteins, both with and without a 3×HA C-terminal tag, were tested in the *N*. *benthamiana* transient expression system. Previously, the chimeric construct Rp1-dp2-D2, with the N-terminus deriving from Rp1-dp2 and the C-terminus from Rp1-D, with a recombination point at 980 AA (Fig. 6A), had been shown to cause HR in transgenic maize [21]. The identical chimeric construct, hereafter named Hd2, induced a strong HR when transiently expressed in *N*. *benthamiana* (Fig. 6A), consistent with the transgenic maize result.

**Fig 7:**
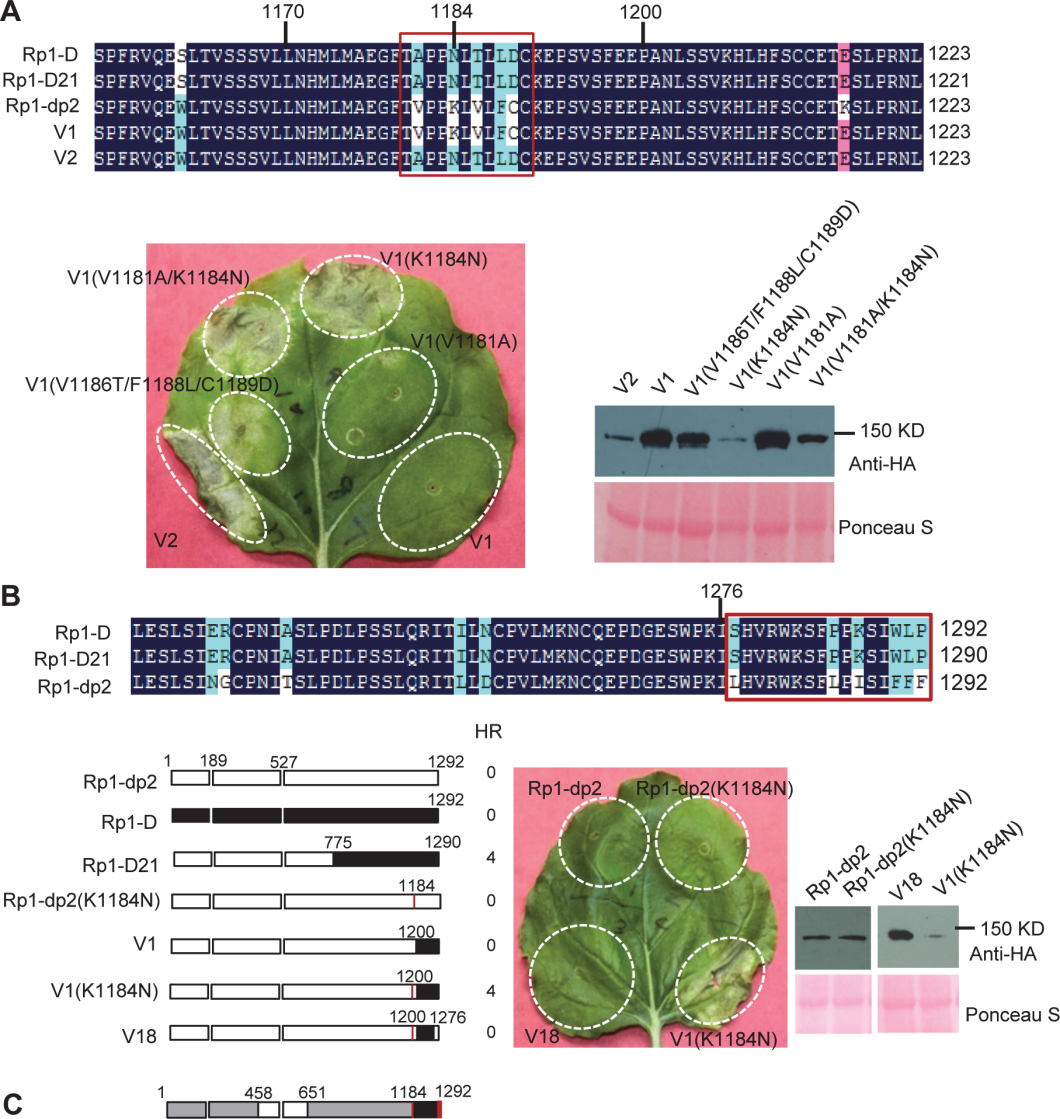
Investigating the functional regions of amino acids (AAs) from 1170–1200 and the C-terminal end of Rp1-D21. (A) Investigating the point mutations in the region of AAs 1170–1200 which are important for inducing hypersensitive response (HR). Top: Multiple sequence alignment of Rp1-D, Rp1-D21, Rp1-dp2, V1 and V2. The black, red and light blue shaded regions represent 100%, 75% and above 50% similarity of the amino acids, respectively. The polymorphic amino acids are boxed, and the position of 1184 (corresponding to 1182 of Rp1-D21) is labeled above the sequences. Bottom left: The HR phenotype of V1, V2 and V1-derived point mutants as indicated. A representative leaf was photographed at 3 days post infiltration (dpi). Bottom right: Total protein was extracted from agro-infiltrated leaves at 30 hours post infiltration (hpi), and anti-HA antibody was used to detect the expression of the fused proteins. Equal loading of protein samples was shown by Ponceau-S staining of Rubisco subunit. (B) Investigating the phenotype conferred by various recombinants and point mutants at the C-terminal end of Rp1-D21. Top: Multiple sequence alignment of the C-terminus of Rp1-D, Rp1-D21 and Rp1-dp2. The polymorphic amino acids are boxed. Bottom left: Schematic diagram of the constructs used. The K1184N mutation is indicated by a red vertical bar in Rp1-dp2(K1184N), V1(K1184N) and V18. Bottom middle: The HR phenotype of the constructs transiently expressed in *N*. *benthamiana*. A representative leaf was photographed at 3 dpi. Bottom right: Total protein was extracted from agro-infiltrated leaves at 30 hpi, and anti-HA antibody was used to detect the expression of the fused proteins. Equal loading of protein samples was shown by Ponceau-S staining of Rubisco subunit. The experiments were repeated three times with the same results. (C) The final model presenting the functional regions important for autoactivity. In this model, white represents regions derived from Rp1-dp2 and black the region from Rp1-D that need to be present in the same construct to result in the autoactive HR in *N*. *benthamiana*. Regions in grey can be derived from either paralog. The combination of AA 458–651 from Rp1-dp2 and AA 1184–1292 (especially N1184 and the last 16 AAs in red vertical bars) from Rp1-D are important for HR induction.

**Fig 8:**
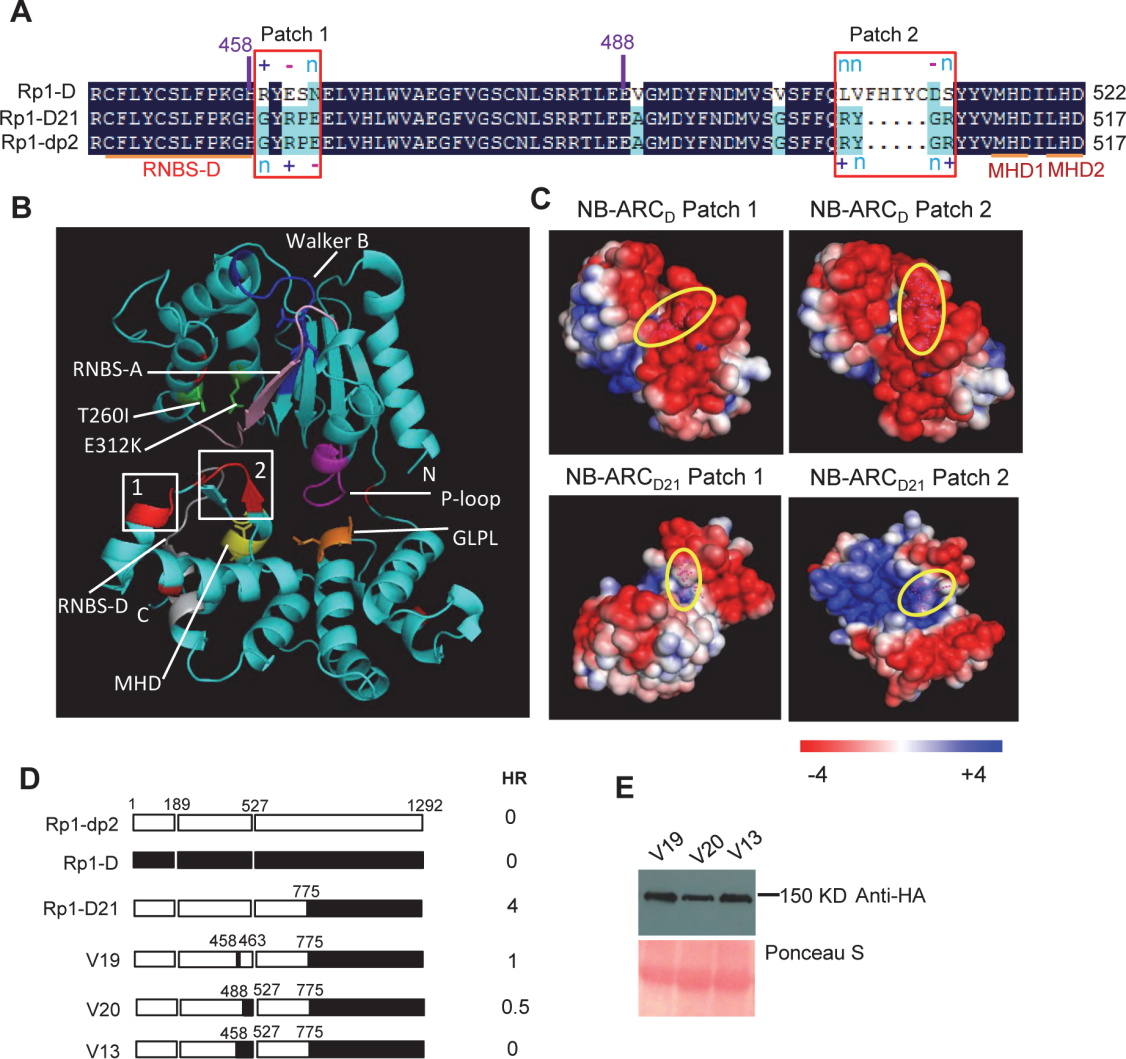
Investigating the function of Patch 1 and 2 from the ARC2 domain for Rp1-D21 autoactivity. (A) Sequence alignment of Rp1-D, Rp1-D21 and Rp1-dp2 in Patch 1 and 2 regions from the ARC domain. Positive (+), negative (-) and neutral (n) charges were labeled. RNBS-D, MHD1 and MHD2 motifs are indicated by orange bars and labeled below the sequences. (B) Homology modeling of NB-ARC_D21_ based on the crystal structure of APAF1 (PDB: 1z6t). The P-loop, MHD, GLPL, RNBS-A, RNBS-D and Walker B motifs are color-coded in magenta, yellow, orange, pink, green and blue, respectively. Two boxed regions (Patch 1 and 2) are the major polymorphic regions between Rp1-D and Rp1-dp2 and are labeled in red. The positions of the suppressor mutations T260I and E312K identified by EMS mutagenesis are indicated in green. The N- and C-termini are indicated as N and C, respectively. (C) The surface electropotential of NB-ARC_D21_ and NB-ARC_D_. The positive and negative charges are indicated in blue and red, respectively. The positions of Patch 1 and Patch 2 are indicated by pink dots in the yellow oval boxes. (D) Schematic diagram and the hypersensitive response phenotype of the constructs indicated. (E) Protein expression was detected by anti-HA antibody at 3 days post-infiltration. Ponceau-S staining of Rubisco subunit was used as loading control. The experiment was repeated three times with the same results.

The results of the transient expression of a series of chimeric proteins are summarized in Fig. 6. The HR phenotype conferred by the constructs fused with 3×HA was largely similar to that conferred by the constructs without any tag (Fig. 6A). All the tagged proteins accumulated to high and broadly comparable levels (Figs. 7 and 8; S6 Fig.).

Constructs V1 through V7 were generated with recombination points between Rp1-dp2 and Rp1-D ranging from AAs 1200 to 488 (Fig. 6A). V1 with a recombination point at AA 1200 did not confer HR while V2 with a recombination point just 30 additional AAs N-terminal to AA 1170 produced a strong HR, indicating that AAs 1170–1200 from Rp1-dp2 are important for auto-inhibition of V1. V4 and V5 (recombination point at 690 and 651, respectively) also produced strong HR phenotype, while V6 and V7 (recombination point 575, 488 respectively) conferred very weak or no HR (rating 1.5 and 0, respectively, Fig. 6A). These results suggest that the AAs 488–651 from Rp1-dp2 are important for the autoactivity of Rp1-D21. Together these data suggest that proteins with a combination of AAs 488–651 from Rp1-dp2 and AAs 1170–1292 from Rp1-D replacing the corresponding sequences of either parental protein are unable to maintain a self-inhibited state.

To test this hypothesis, we investigated whether swapping the N-terminal region (CC, NB-ARC or AAs 1–488) in Rp1-D21 affected its autoactivity. We generated 8 more chimeric constructs in which we exchanged the Rp1-dp2-derived N-terminal parts of Rp1-D21 with the corresponding parts from Rp1-D (Fig. 6B). Construct V8, in which the CC-NB-ARC (AAs 1–527) of Rp1-dp2 was replaced with the corresponding Rp1-D sequence, did not confer an HR. Similar results were obtained by exchanging the NB-ARC domain in V9. However, V10, exchanging the CC domain of Rp1-D into Rp1-D21 conferred a strong HR (Fig. 6B). These results suggest that the ‘parental origin’ of the CC domain (AAs 1–189) in Rp1-D21 plays only a minor or no role in its autoactive phenotype. V11 and V12 were generated by replacing AAs 190–370 or 190–458 (NB-ARC1 plus part of ARC2, respectively) of V10 with the corresponding Rp1-D sequence. They differed from V10 by only 2 and 5 single amino acid polymorphisms (SAAPs), respectively. V11 conferred a slightly weaker HR than V10 and the HR conferred by V12 was weaker still (Fig. 6B). Construct V13, of which the CC-NB-ARC domain was ‘reciprocal’ of V12, did not confer HR, confirming that AAs 458–527 (ARC2 region) from Rp1-dp2 were important for the autoactivity of Rp1-D21 (comparing V13, V12 and V8). Constructs V14 and V15 were generated by exchanging AAs regions 690–775 and 651–775 respectively of V10 with the corresponding Rp1-D sequence. Both constructs showed strong HR (Fig. 6B), similar with the corresponding AAs exchanges in Rp1-D21 (constructs V4 and V5 in Fig. 6A). V16 and V17 were ‘reciprocals’ of Rp1-D21 and V3, respectively and did not confer an HR phenotype (Fig. 6C).

In summary, this set of experiments indicated that the combination of AAs 458–651 from Rp1-dp2 and AAs 1170–1200 from Rp1-D is central to the deficiency of self-inhibition of Rp1-D21.

### Defining specific regions (amino acids) important for Rp1-D21 autoactivity

There are only 17 single amino acid polymorphisms (SAAPs) between AAs 1170–1292 of Rp1-D and Rp1-dp2, and only 5 SAAPs in the AAs 1170–1200 region that differentiate constructs V1 and V2 (Figs. 6A and 7A). However, V2 was autoactive, while V1 was not (Fig. 6). To analyze which SAAP is important for the self-inhibition of Rp1 proteins, we generated several mutants based on these 5 SAAPs in the autoinhibited construct V1, including V1(V1181A), V1(K1184N), V1(V1181A/K1184N) and V1(V1186T/F1188L/C1189D). When transiently expressed in *N*. *benthamiana*, V1(V1181A) was not autoactive, while V1(K1184N) and V1(V1181A/K1184N) produced a strong HR (rating 4), and V1(V1186T/F1188L/C1189D) a weak HR (rating 2; Fig. 7A). The results indicated that residue K1184 from Rp1-dp2 plays an important role in self-inhibition, and the residues V1186/F1188/C1189 play a minor role. Interestingly, one of the Rp1-D21 suppressor EMS mutants had a mutation at residue 1180 (P to S), only 2 AAs away from N1182 in Rp1-D21 (equivalent to N1184 in Rp1-D), emphasizing the importance of this region for regulating the activity of the Rp1 proteins.

To investigate whether mutating K1184 was sufficient to convert Rp1-dp2 into an autoactive NLR, we generated Rp1-dp2(K1184N). However, this single point mutation was not able to ‘activate’ Rp1-dp2 (Fig. 7B). This result suggests that additional SAAPs in the region of AAs 1200–1292 are also important for self-inhibition of Rp1-dp2. There are 12 additional SAAPs in the last 92 AAs between Rp1-D and Rp1-dp2 and of these six are found in the last 16 AAs (Fig. 7B). Therefore, we constructed the chimeric protein V18 by replacing the C-terminal 16 AAs in V1(K1184N), the autoactive V1 protein, with the equivalent AAs from Rp1-dp2. Interestingly, this chimeric protein V18 lost the ability to induce HR (Fig. 7B), demonstrating that the very C-terminal 16 AAs are very important for the self-inhibition of the Rp1 proteins.

In conclusion, these experiments further defined the combination of AAs required for the autoactivity of Rp1-D21: a combination of AAs 458–651 from Rp1-dp2 and AAs 1184–1292 (especially N1184 and the last 16 AAs) from Rp1-D are central to the autoactivity (see model in Fig. 7C).

### Homology modeling of the NB-ARC from Rp1-D and Rp1-D21 reveals novel structural elements required for HR

As shown in Fig. 6B (construct V13), AAs 458–527 from Rp1-dp2 were very important for the autoactivity of Rp1-D21. There are two major regions of polymorphism, which we termed Patch 1 and 2, between Rp1-D and Rp1-D21 within the ARC2 sub-domain, from AAs 458–527 (Figs. 1 and 8). Homology modeling based on the crystal structure of the NB-ARC domain of the ADP-bound human apoptosis regulator APAF1 (Protein data bank entry 1z6t; [40]) predicted that Patch 1 and 2 were surface-exposed (Figs. 8A and 8B). Surface electropotential predictions indicated that the surfaces of both patches in Rp1-D were mostly negatively charged, while for Rp1-D21 Patch 1 was slightly positively charged and Patch 2 was mostly positively charged (Fig. 8C). To investigate whether Patch 1 and 2 played roles in the autoactivity of Rp1-D21, we constructed another two chimeric proteins, V19 and V20, by replacing AAs 458–463 (Patch 1) and 488–527 (Patch 2) in Rp1-D21 with the corresponding AAs from Rp1-D, respectively (Fig. 8D). Both V19 and V20 conferred a greatly reduced HR compared to Rp1-D21, while V13 (containing Patch 1+2 from Rp1-D) did not cause HR (Fig. 8D), suggesting that these patches have an additive effect on the autoactivity of Rp1-D21.

### Intra- and inter-molecular interactions of Rp1 proteins

**Fig 9:**
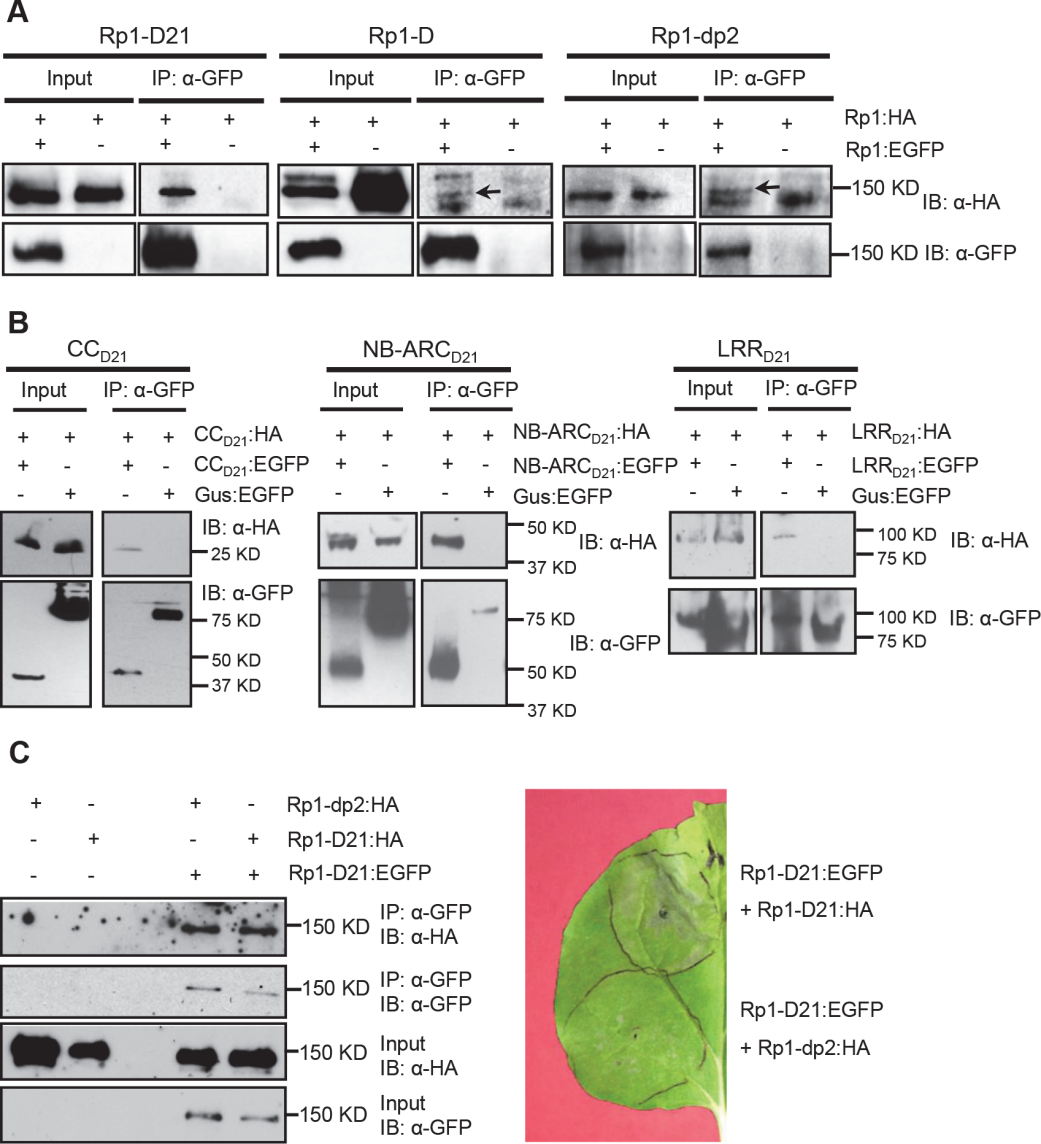
Investigation of self-association of full length Rp1-D21, Rp1-D, Rp1-dp2, the separate domains of Rp1-D21, and the interaction of full length Rp1-D21 with Rp1-dp2. (A) Self-association of 3×HA- and EGFP-tagged Rp1-D21 (left panel), Rp1-D (middle panel) and Rp1-dp2 (right panel). Arrows indicate the target bands. (B) Self-association of CC_D21_, NB_D21_ and LRR_D21_. (C) Left: Co-IP after co-expression of Rp1-D21 and Rp1-dp2. Right: HR induced by co-expression of Rp1-D21:EGFP with Rp1-D21:HA compared to HR induced by co-expression of Rp1-D21:EGFP with Rp1-dp2:HA. EGFP- and 3×HA-tagged constructs were transiently co-expressed in *N*. *benthamiana* and samples were collected at 30 hours post infiltration for Co-IP assay. Protein extract was immunoprecipitated by anti-GFP microbeads and detected by anti-GFP and anti-HA antibodies. The experiments were repeated three times with the same results.

Self-association has been observed in both CNL (eg. barley MLA, Arabidopsis RPS5) and TNL (eg. tobacco N, flax L6) proteins and is important for the activity of NLRs [38,41,42,43]. To test whether Rp1-D21, Rp1-D or Rp1-dp2 could self-associate, we transiently co-expressed EGFP- and 3×HA-tagged proteins in *N*. *benthamiana* and performed co-immunoprecipitation (Co-IP) analyses. We observed that full-length Rp1-D21, Rp1-D and Rp1-dp2 all self-associated (Fig. 9A). We further showed that all three of the domains (CC_D21_, NB-ARC_D21_ and LRR_D21_) of Rp1-D21, were able to self-associate (Fig. 9B).

To further investigate whether Rp1-D or Rp1-dp2 could form heteromers with Rp1-D21, we transiently co-expressed Rp1-D21:EGFP and 3×HA-tagged Rp1-D or Rp1-dp2 proteins in *N*. *benthamiana* and performed Co-IP analyses. We found that Rp1-dp2 interacted with Rp1-D21 (Fig. 9C). We did not detect interaction between Rp1-D and Rp1-D21, which might have been due to the low expression of Rp1-D (Fig. 3B). Consistent with the interaction data, we observed that Rp1-dp2 could partially suppress Rp1-D21-induced HR (Fig. 9C).

### ARC2_D21_ is important for the interaction of NB-ARC_D21_ with CC_D21_

**Fig 10:**
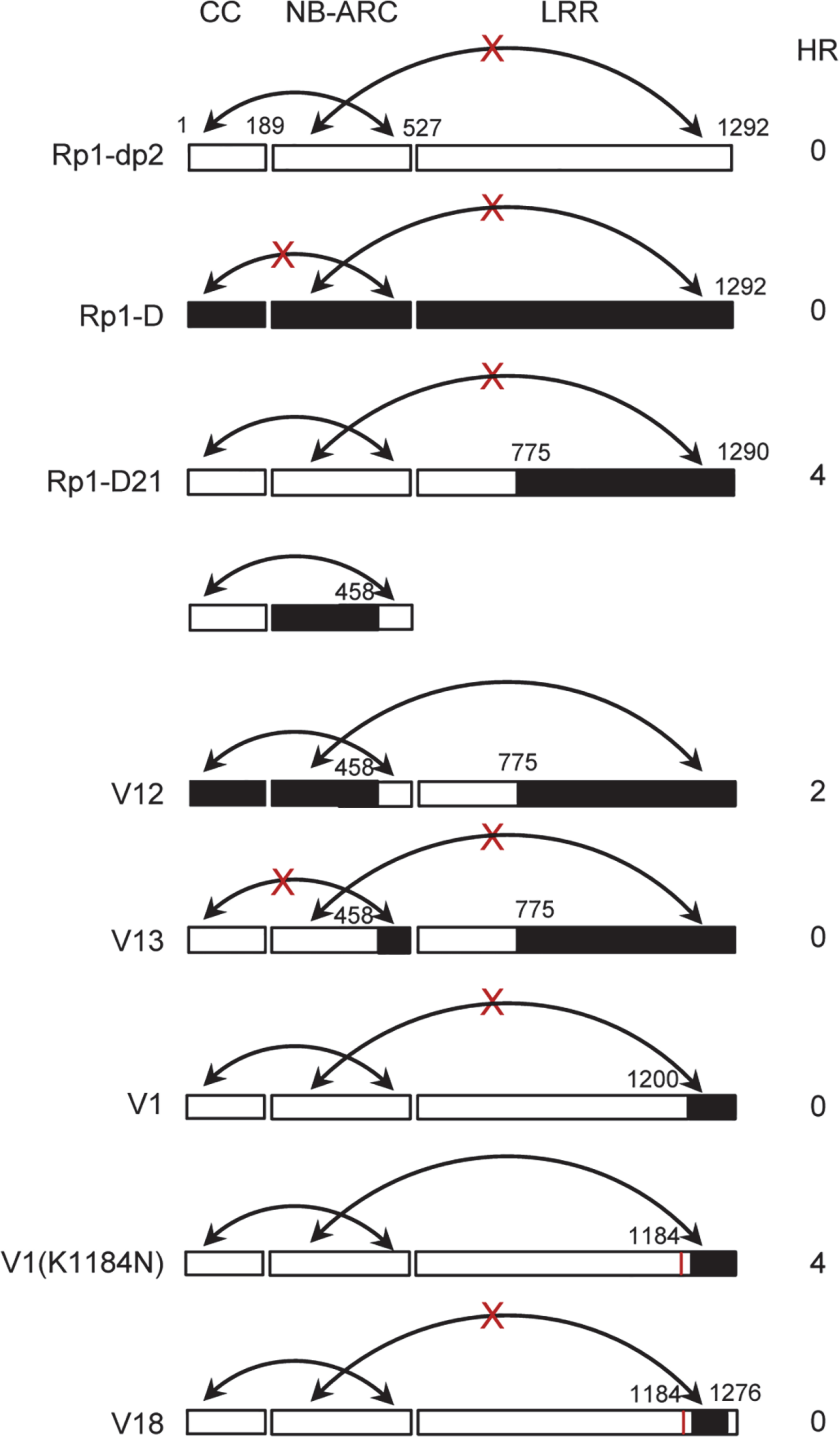
Schematic diagram of the intra-molecular interactions and HR phenotype of the constructs indicated, summarizing the data shown in S7 Fig. in which the various domains were co-expressed and co-immunoprecipitated *in trans*. The gene structure has been divided into CC, NB-ARC and LRR domains. The amino acid position of the recombination site of each construct was indicated above the construct. For each construct, the strength of the hypersensitive response (HR) resulting from transient expression of each full length molecule in *N*. *benthamiana* was scored on a 0 (no HR) to 5 (strong HR) scale. The ability to form or not to form intra-molecular interactions among different domains was indicated by arrows or arrows with crosses, respectively.

To investigate whether different intra-molecular interactions are correlated with the different activities of Rp1-D21 and Rp1-D, we co-expressed pair-wise combinations of different domains in *N*. *benthamiana* and analyzed by Co-IP. We observed that NB-ARC_D21_ interacted with CC_D21_, but not with LRR_D21_ or LRR_dp2_ (Fig. 10; S7A–S7B Fig.). Additionally, we did not observe interactions between NB-ARC_D_ and CC_D_ or between NB-ARC_D_ and LRR_D_ under our conditions (Fig. 10; S7A–S7B Fig.).

Since the CC and the NB-ARC domains in Rp1-D21 and Rp1-D have different interactions, we investigated the regions required for this interaction. As noted above, the ARC2 domain (including Patch 1 and 2) is the major difference in the NB-ARC domain between Rp1-D21 and Rp1-D. In order to test whether this region affects the interaction between the CC and NB-ARC domains, we performed a Co-IP assay with transiently co-expressed CC_D21_/CC_D_ and NB-ARC_V12_/NB-ARC_V13_, and observed that NB-ARC_V12_ interacted with both CC_D_ and CC_D21_, while NB-ARC_V13_ did not interact with CC_D21_ (Fig. 10; S7A Fig.). The results suggested that ARC2_D21_ (AAs 458–527 from Rp1-dp2) is important for the interaction between CC and NB-ARC domains. In other words, ARC2_D_ can suppress the interaction.

We also observed that LRR_D21_ interacted with NB-ARC_V12_, but not NB-ARC_V13_ (Fig. 10; S7B Fig.), suggesting that NB-ARC1_D_ is required for the interaction between NB-ARC and LRR_D21_, which is consistent with previous report that ARC1 is required for binding to LRR of potato CNL protein, Rx [44].

### N1184 and the last 16 AAs from Rp1-D are required for the interaction between NB-ARC_D21_ and LRR_V1(K1184N)_

To determine whether, as suggested by the recombination studies, N1184 and the C-terminal 16 AAs are involved in the intra-molecular interaction, we performed Co-IP assays with NB-ARC_D21_ and LRR_V1_, LRR_V1(K1184N)_ or LRR_V18_ and observed that NB-ARC_D21_ interacted with LRR_V1(K1184N)_, but not with LRR_V1_ and LRR_V18_ (Fig. 10; S7C Fig.), indicating that N1184 and the last 16 AAs from Rp1-D are required for the interaction between NB-ARC_D21_ and LRR_V1(K1184N)_.

### The effects of CC domain EMS mutants on CC_D21_-induced HR

We investigated the effect of the five CC domain missense EMS mutations on CC_D21_-induced HR. Consistent with the results from full length proteins, CC_R125W_ abolished HR induction and CC_L89F_ and CC_S108F_ reduced HR, while CC_H59Y_ had no obvious effect compared to CC_D21_ (Table 1). Surprisingly, CC_D82N_ induced stronger HR than CC_D21_, while CC_D82N_ in the full length Rp1-D21 did not induce HR (Table 1).

Co-IP experiments were performed to explore whether the CC domain mutations affect the self-association of CC_D21_. CC_D82N_ but not the other four point mutants reduced the strength of CC_D21_ self-association (S8A Fig.). To further test whether the mutations affect the interaction between CC_D21_ and NB-ARC_D21_, we chose CC_D82N_ and CC_R125W_, two mutants that completely abolished HR in full length, to test the inter-domain interaction. We found that both CC_D82N_ and CC_R125W_ greatly reduced the interaction between CC_D21_ and NB-ARC_D21_ (S8B Fig.).

## Discussion

### A genetic screen identifies intragenic Rp1-D21 suppressor mutants

While 10 of the 12 Rp1-D21 loss of function EMS mutants isolated from maize also reduced or abolished the HR phenotype when transiently expressed in *N*. *benthamiana*, two, H59Y and G850D, did not. It is possible that these two mutations reduced the activity of Rp1-D21 to a level below the threshold for signaling in maize, but not in *N*. *benthamiana* due to the higher expression.

One mutation (D82N) was in the conserved EDVID motif. Previous reports have indicated an important role for the EDVID motif in NLR function. In Rx, the EDVID motif mediates the intra-molecular interaction between the CC and the NB-ARC-LRR domains [30,45] and the residues flanking the EDVID motif affect inter-molecular interactions with RanGAP2, which is required for Rx function [30,46,47]. In this study, we found that D82N abolished HR induced by full length Rp1-D21, but not by CC_D21_ (Tables 1 and 2), similar to what was observed for a mutation in the EDVID motif of MLA10 [27]. We further showed that CC_D82N_ and CC_R125W_ reduced the interaction with NB-ARC_D21_ (S8B Fig.). The “mousetrap model” for NLR activation [48,49] suggests that, like a mousetrap, the NLR protein must be ‘set’ in a primed state, ready to be activated. The apparent paradox that the D82N mutation causes loss of autoactivity in full length Rp1-D21 but not in the CC domain alone may be explained by the reduced association of CC_D21_ and NB-ARC_D21_ and the consequent loss of ability to set up this initial primed state in the full-length protein. It is also likely that the mutations in the CC domain might disrupt the inter-molecular interactions of Rp1-D21 with other co-factors.

Three of the missense mutations were within or next to the ADP binding pocket of the NB-ARC domain when the NB-ARC was modeled onto the APAF-1 structure; T260I was close to the RNBS-A motif, E312K was next to Walker B motif and P398L was in the GLPL motif (Table 1; Figs. 1 and 8B), suggesting that these mutations might change the ADP binding state. Consistent with our results, important roles for the RNBS-A, Walker B and GLPL motifs on modulating HR have been reported in other NLRs [14,42,44]. Mutations in the Walker B and GLPL motifs can also affect the intra-molecular interaction between CC and NB-ARC-LRR domains as evidenced in Rx [30]. Thus, we infer that the three mutations in the NB-ARC domain might also affect the interaction between CC_D21_ and NB-ARC_D21_.

Finally, four of the suppressor mutations (S737L, S794F, G850D and P1180S) were in the LRR domain (Fig. 2B). Loss-of-function mutations in the LRR domain have been reported in several NLRs [16,50,51,52,53,54]. According to our model (see below), the mutations likely abolish the ability of the LRR_D21_ domain to destabilize the interaction between NB-ARC_D21_ and CC_D21_.

### Rp1 activity is P-loop- and MHD-dependent

Mutations from H (histidine) to A (alanine) or D (aspartate) to V (valine) in the highly conserved MHD domain result in constitutive activity of multiple NLRs from multiple species [17,27,37,41,55,56]. In the Flax NLR, M, ADP is bound to wild-type NB-ARC domain, while ATP is bound to the autoactive MHD mutant M(D555V) [15], providing direct evidence that the inactive “off” NLR binds ADP while the active “on” NLR binds ATP. It appears therefore that the MHD motif is important for maintaining the NLR protein in its appropriate state of activity.

Rp1-dp2 and Rp1-D as well as several other CNLs, but not TNLs, have two MHD motifs [17,37]. We have termed these motifs MHD1 and MHD2 (Fig. 4A). MHD1 is more conserved and is the functional MHD motif for many NLRs as defined in previous studies [15,17,27,38,56]. The aspartate (D) in MHD2 is quite widely conserved throughout most CNLs [37]. Of the CNLs that contained two MHD motifs [37], the effects of mutations in the MHD1 domain only have been reported for tomato NLR Mi-1.2(H840A) and Mi-1.2(D841V). Both mutations activate these proteins [34]. The possible function of the MHD2 domain has recently been examined in rice CNL-RGA5, but no functional effect was observed [57]. Transient expression of Rp1-dp2(D517V) and of V1(D517V) and V16(D522V), in which the MHD2 was mutated, conferred autoactive HR, while no MHD1 mutation had this effect (Figs. 4B and 4C; S3 Fig.). It is possible that the MHD2 rather than the MHD1 domain is functional in Rp1-dp2, or that the MHD1 and MHD2 domains coordinate the activity. This is the first report showing the functional significance of the MHD2 domain of a CNL for its activity.

The P-loop motif in the NB-ARC domain regulates nucleotide binding. P-loop mutations abolish the ability to confer disease resistance or HR induction in multiple NLR proteins [14,15,27,34,38,39,56]. As expected, the HR induced by Rp1-D21 and the MHD2 mutant Rp1-dp2(D517V) was abrogated by the introduction of a P-loop mutation (Fig. 4D), indicating that the activity of the Rp1 proteins is P-loop dependent.

### Two minimum functional regions in the NB-ARC and the LRR domain are important for regulating Rp1 protein activity

There are several examples in the literature of recombinations between the TIR-NB-ARC or CC-NB-ARC and LRR domains of different NLRs resulting in autoactive proteins. The recombination of CC-NB-ARC from Gpa2 with LRR from Rx1 produces a gene conferring autoactive HR; The combination of Gpa2-ARC2 and the first two repeats of Rx-LRR region is essential for autoactivity [36,44]. Domain swaps between RPS5 and RPS2 [29] and between Mi-1.1 and Mi-1.2 [58] also gave rise to genes conferring an autoactive phenotype. In contrast to these artificially-constructed autoactive NLRs characterized in transient assays, Rp1-D21 is an autoactive protein that occurred via recombination and was identified in its endogenous genetic background. To identify the precise structural requirement for its activity, we performed a systematic structural analysis of Rp1-D21 using a set of artificial recombinants between the two ‘parental’ alleles (Fig. 6) and showed that the combination of AAs 458–651 (the ARC2 and N-terminus of the LRR region) from Rp1-dp2 and the C-terminal LRR region (especially N1184 and the C-terminal 16 AAs) from Rp1-D was critical for the autoactivity of Rp1-D21. This combination either destabilized the Rp1-D21 intra-molecular interactions that cause the inactive resting state, or stabilized interactions resulting in the active state (see below). In other words, these two regions appear to be important for maintaining the parental proteins in an inactive ‘resting’ state. Thus, in light of the “mousetrap” model [48], this region of the Rp1 family NB-ARC and LRR domains is involved in a precarious autoinhibiting conformation that is easily broken by alterations including exchange of a very small C-terminal region of the LRR domain, or presumably, in a wild-type context by the action of the cognate effector protein. It seems evident that the NB-ARC and LRR domains within each NLR must co-evolve to maintain the NLRs in a suitably auto-inhibited resting state in the absence of pathogen infection while maintaining the ability to respond to the cognate effector via intra-molecular interactions [36,38,55], but that precise mechanisms of auto-inhibition and activation may vary between NLRs.

### The role of Patch 1 and 2 in the ARC2 domain

The ARC2 domain is important for function in several NLRs [36]. We identified two major polymorphic regions localized in ARC2 that differentiated the NB-ARC_D21_ and NB-ARC_D_ domains, Patch 1 and Patch 2, and showed that they are important for the autoactivity of Rp1-D21 (Figs. 1 and 8). Replacing either Patch 1 or Patch 2 of Rp1-D21 with the region from Rp1-D almost completely suppressed the autoactive phenotype (constructs V13, V19 and V20; Fig. 8D). In agreement with our structural modeling, these two patches were surface-exposed and carried largely opposite charges in NB-ARC_D21_ compared to NB-ARC_D_ (Fig. 8B). The prevailing model for the activation of NLRs is that the off state binds ADP while the on state binds ATP [36,59]. Patch 2 is located three AAs N-terminal to the MHD1 motif and is localized in the exposed surface next to the nucleotide binding pocket [40], suggesting it might affect the state of nucleotide binding. Patch 1 is adjacent to a conserved RNBS-D motif in the ARC2 domain (Figs. 1 and 3A). Mutations in or next to the RNBS-D motif of PM3, RPM1 and Rx affected their function [16,36,45,55]. Thus, the sequence differences of Patch 1 between NB-ARC_D21_ and NB-ARC_D_ might also affect the protein activity through disturbing the function of the RNBS-D motif.

Interestingly, we found that ARC2_D21_ is critical for the interaction between NB-ARC_D21_ and CC_D21_ or CC_D_ (Fig. 10, compare V13 with V1 and Rp1-D with V12, and see S7 Fig.). This is, to our knowledge, the first demonstration of the role of ARC2 in determining NB-ARC and CC interaction in plant NLRs. Our modeling data also suggests why we detected interaction between NB-ARC_D21_ and CC_D21_ but not between NB-ARC_D_ and CC_D_ (Fig. 10; S7 Fig.). In the CC domain, the side chain of the EDVID motif of CNLs is largely negatively charged, and this motif is thought to mediate intra-molecular interactions of the CC domain with the NB-ARC-LRR domain of Rx [30,60]. In Rp1 proteins, the negatively charged EDVID and the positively-charged ARC2_D21_ are apparently very important for the interaction between CC_D21_ and NB-ARC_D21_ while the ARC2_D_ is more negatively charged (Fig. 8C) and thus may not interact as strongly with the CC. Interestingly, we observed that AAs 190–260 from the NB domain were sufficient to suppress CC_D21_-induced HR (S4 Fig.), suggesting that ARC2_D21_ is not the only region which can regulate Rp1-D21 autoactivity.

### The role of different Rp1-D21 domains in autoactivity

CC_D21_ and CC_D_ domains alone were sufficient to induce HR when fused with EGFP, but not with 3×HA or on their own (Fig. 5; S1 Fig.). This phenomenon of “tag-dependent activity” has been observed in other NLR studies [30,61]. The functional domains required for HR induction vary in different NLR proteins. The CC domain alone is sufficient for the HR phenotype triggered by MLA10, and also by NRG1 and ADR1, which contain atypical CC domains [28,41], while the NB-ARC domain of Rx can trigger HR [30], and for RPS5 the CC-NB-ARC is sufficient [29].

The fact that transient expression of CC-NB-ARC_D21_ or CC-NB-ARC_D_ did not induce HR suggested that their respective NB-ARC domains repressed the signaling by the CC domain. Since full length Rp1-D21 induced HR and full length Rp1-D did not (Fig. 3), we inferred that the LRR_D21_ or LRR_D_ domains are structurally incompatible with, respectively, repression of CC_D21_ autoactivity or with CC_D_ autoactivity (Fig. 5). While interaction between the LRR and NB-ARC domains was detected in some combinations (e.g., see proteins V12, V1(K1184N) in Fig. 10), we did not detect any interaction between the LRR and NB-ARC domains of Rp1-D, Rp1-dp2 or Rp1-D21 *in trans* (Fig. 10; S7 Fig.). It is possible though that these domains interact in the full length context, or that their interactions are weak or transient and cannot be detected in our Co-IP conditions. Consistent with this, LRR_D21_ was unable to alter the suppression of CC_D21_ autoactivity by NB-ARC_D21_ *in trans* (Table 2). However, we cannot exclude the possibility that the lack of suppression *in trans* was due to the relatively low expression of LRR_D21_ (Table 2; Fig. 5B). A similar finding of *cis* but not *trans* interaction/autoactivity was reported for potato Rx and autoactive MHD mutants of tomato Mi-1.2 [34,44,62]. It is notable also that the CC-NB-ARC fusion from MLA10 and Rx can still trigger HR [27,30], but not in Rp1-D and Rp1-D21.

LRR domains have multiple reported roles in NLR activation. They are essential for the activation of the NLRs Rx and Mi-1.2 [34,62]. In the case of RPS5, the first four LRRs are the minimum region sufficient to inhibit the autoactive phenotype conferred by the CC-NB-ARC domain [29]. Conversely, the activation of RPS5 in response to disease requires the entire LRR domain [29]. Our analyses of Rp1-D21 C-terminal deletion constructs and LRR domain swap data (Fig. 7B; S5 Fig.) further confirmed the importance of the C-terminal LRR domain in regulating the activity of the Rp1 proteins. Similarly, the importance of C-terminal LRR domain has also been observed in flax TNL, L proteins [37].

The interaction patterns observed between NB-ARC and LRR from V1, V1(K1184N) and V18 suggested that K1184N and the last 16 AAs from Rp1-D are required for the inter-domain interaction (Fig. 10; S7 Fig.). No interaction was detected between the NB-ARC and LRR domains from V1 or between those from V18 when they were expressed *in trans*, but a *trans-*interaction was detected between these domains using the V1(K1184N) construct. Assuming that these interactions are maintained in the full length proteins, this result implies that the ability of V1(K1184N) to induce HR is due to the interaction of the LRR with the NB-ARC, which apparently is then locked into an activated state allowing CC-dependent HR. This also requires the C-terminal 16 amino acids of the Rp1-D21 LRR domain (Fig. 10; S7 Fig.). A further inference is that the C-terminal 16 AAs and N1182 in Rp1-D21 (corresponding to N1184 in Rp1-D) are crucial for the inhibition of NB-ARC activity that is required for CC-dependent HR.

### A model for the autoactivity of Rp1-D21

**Fig 11:**
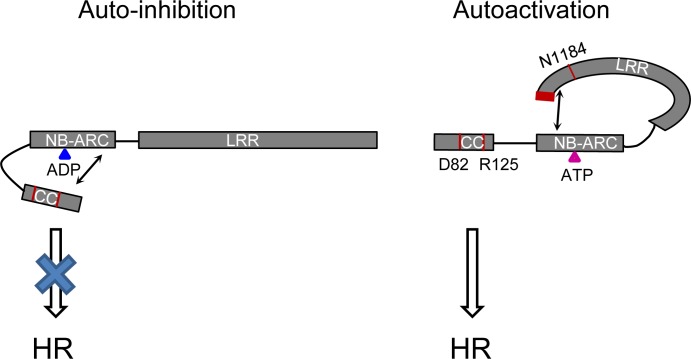
Model of the intra-molecular interactions controlling auto-inhibition or auto-activation in Rp1 proteins. In the auto-inhibited state (left), the NB-ARC domain inhibits CC-induced HR via intra-molecular interaction involving the ARC2 domain. In the auto-activated state (right), the LRR suppresses an inhibitory effect of the NB-ARC on the CC through direct interaction with the NB-ARC which destabilizes the CC/NB-ARC interaction. Blue and pink triangles indicate ADP and ATP, respectively. Arrows indicate intra-molecular interactions. The positions of amino acids (D82, R125, N1184 and the C-terminal 16 amino acids) which are important for activity are labeled in red. The rationale behind the models is explained in the main text.

Alternate “on” or “off” states mediated through intra-molecular interactions are thought to be one of the major mechanisms regulating NLR activity [51,62]. For example, intra-molecular interactions were detected between CC and NB-ARC-LRR, and CC-NB-ARC and LRR domains of Rx in the absence but not in the presence of its cognate effector, the PVX coat protein [62]. Similarly the differentially-activated states of Rp1-D, Rp1-dp2, Rp1-D21 and our additional recombinant proteins are likely due to the specific intra-molecular interactions found within each protein. While Rp1-D21 and V3 confer HR, their reciprocal constructs, V16 and V17, do not (Fig. 6C), indicating that the autoactivity of the recombinant protein is triggered by specific combination of sequences from Rp1-dp2 and Rp1-D. Our model (Fig. 11) explaining the intra-molecular interactions underlying the activity of the Rp1 proteins is as follows:

1. *Self-association and heteromer formation between Rp1 proteins are important for their activity*.
  Self-association of NLRs has been shown to be important for activity in other cases [38,41,42,43]. While we have not been able to show that this is the case for Rp1 proteins, we have shown that Rp1-D21, Rp1-D, Rp1-dp2 and each of the three major domains, CC_D21_, NB-ARC_D21_, LRR_D21_ can self-associate (Fig. 9). Based on these related studies, we propose that this self-association is important for function.
  Heteromers formed by different NLRs have been shown to be important for NLR activity [57,63]. We found that Rp1-dp2 and Rp1-D21 physically interacted and that their co-expression suppressed Rp1-D21-induced HR, presumably due to their physical interaction (Fig. 9C). This is also consistent with our previous study in which we identified a quantitative trait locus mapping directly to Rp1 which regulated the severity of Rp1-D21-induced HR in maize [26].
2. *The CC domain is the signaling competent region of the Rp1 proteins*.
  This is demonstrated by the ability of CC expressed alone to induce HR (Fig. 5).
3. *The NB-ARC domain inhibits CC-induced HR in Rp1-D21 via intra-molecular interactions involving the ARC2 domain*.
  In Rp1-D, NB-ARC_D_ can inhibit CC_D_-induced HR *in cis*, but not *in trans* (Fig. 5A; Table 2). Though we did not detect direct physical interaction between NB-ARC_D_ and CC_D_, the fact that NB-ARC_D_ inhibits HR caused by CC_D_ *in cis* suggests that there is inhibitory interaction between the CC and NB-ARC domains in both Rp1-D and Rp1-D21. Data from the recombination (Fig. 6) and Co-IP experiments (Fig. 10; S7 Fig.) indicate that the ARC2 domain is important for controlling both activity and intra-molecular interactions of Rp1 proteins. We propose that the intra-molecular CC/NB-ARC interactions in which ARC2 domain plays a critical role are important for setting up a ‘primed’ state as proposed under the “mousetrap model” for NLR activation [48,49].
4. *In* Rp1-D21 *and the other autoactive recombinant proteins*, *the LRR destabilizes the CC/NB-ARC interaction*, *leading to auto-activation*.
  The best evidence for this comes from the comparison of the interaction patterns of V1, V1(K1184N) and V18 in Fig. 10. These proteins differ by only 1 and 6 AAs, respectively. In V1(K1184N) the LRR and NB-ARC domains interact *in trans*. This is correlated with the ability of the corresponding full length protein to confer HR in the transient expression system, since neither V1 nor V18 confer HR and their respective LRR domains do not interact with the shared NB-ARC. Consistent with these data, truncation of the C-terminal 34 AAs of Rp1-D21 eliminates autoactivity (see also #6, below). Thus, 7 AA changes from Rp1-D in Rp1-D21 in a protein otherwise derived from Rp1-dp2 are sufficient to de-stabilize the LRR’s ability to inhibit ectopic NB-ARC activity and consequent CC-dependent HR.
  Although we did not observe Co-IP between LRR_D21_ and NB-ARC_D21_, we propose, for reasons detailed above, that there is an interaction between these two domains *in cis* but not *in trans*. This would be consistent with our data that NB-ARC-LRR_D21_ is unable to inhibit CC_D21_ signaling when present *in trans* (Table 2).
5. *Destabilization of the CC/NB-ARC inhibitory interaction allows for the exchange of bound ADP for ATP in a P-loop*, *RNBS-A*, *Walker B*, *GLPL and MHD2/ARC2-dependent manner*.
  Mutants in or next to P-loop, RNBS-A, Walker B, GLPL and ARC2 lose the ability to confer HR (Table 1; Fig. 4; S2 Fig.).
6. *The autoactivated state of Rp1-D21 and various other derived autoactive recombinants in which the CC/NB-ARC interaction is destabilized*, *depends upon the combined presence of AAs 458–651 (Patches 1 and 2 in ARC2 and the N-terminal LRR region) from Rp1-dp2 and of the C-terminal LRR region (especially N1184 and the last 16 AAs) from Rp1-D*.
  The data for this is discussed above and detailed in Figs. 6 and 7.

While we have not identified a cognate effector of Rp1-D and do not know how it is detected, we hypothesize that its presence is necessary for activating Rp1-D via direct or indirect interaction with the LRR domain. This model is based both upon the data presented here and a review of the related literature. While plant NLR resistance genes share similar structures and appear to perform very similar functions, it is clear that the molecular mechanisms underlying their function and their appropriate activation, while sharing certain similarities, vary substantially. This variation likely reflects the intimate and unique co-evolutionary processes each NLR has undergone both with respect to the interactions among their different domains and with their cognate pathogen effector proteins, and/or the host targets or decoys of those effectors. The molecular mechanisms underlying the auto-inhibition of the maize NLR common rust resistance protein Rp1-D and its autoactive derivative Rp1-D21 show both broad similarities to and distinct differences from what is known of other NLRs. Furthermore, we have identified several unique, or previously un-observed features, including: the AAs required for HR induction identified through EMS mutagenesis screening; the functional characterization of MHD2 motif; the involvement of the ARC2 domain in the interaction of the NB-ARC domain with the CC domain; the observation that N1184 and the C-terminal 16 AAs are involved both in the LRR/NB-ARC physical interaction and in regulating activity.

Recently, several NLR genes that have been transferred between plant species were demonstrated to still confer expected disease resistance specificities without obvious fitness effects, eg. Arabidopsis *RPS4* (*Resistance to Pseudomonas syringae 4*), *RRS1* (*Resistance to Ralstonia solanacearum 1*) and barley *MLA1* [64,65]. Genetic manipulation of *Rx* indicates stepwise artificial evolution can be used to reduce the costs associated with disease resistance of NLRs [66]. Rp1-D21 is an autoactive mutant conferring nonspecific response to multiple maize rust species, including *P*. *sorghi* and *P*. *triticina* [19,21]. Presumably the resistance conferred by *Rp1-D21* might extend beyond rusts to other biotrophic and hemi-biotrophic pathogens, however, the severe growth penalties associated with the expression of this gene make its application in agricultural production impractical. In conjunction with suitable promoters, it may be possible to engineer maize or even other plants with some of the chimeric constructs characterized here that confer a weaker HR phenotype to achieve an elevated disease resistance with fewer fitness consequences.

## Materials and Methods

### Plant materials and growth conditions

Wild type *N*. *benthamiana* plants were grown at 23°C with a cycle of 16 hrs light/8 hrs dark. Maize line *Rp1-D21*-H95 [25] was used to isolate the genomic DNA sequence of *Rp1-D21*. To generate material for EMS mutageneis, *Rp1-D21* was first introgressed into A632 by 7 backcrosses (BC7). A few of these BC7 *Rp1-D21* heterozygous plants were self-pollinated to generate *Rp1-D21* homozygotes in the A632 background which were identified on the basis of their enhanced *Rp1-D21* severity compared to the heterozygotes.

### EMS mutagenesis screening

Pollen from the homozygous *Rp1-D21* plants in an A632 background was collected and treated with EMS for 45 min before using it to fertilize the ears of an inbred line H95. The resulting M1 population was all heterozygous for *Rp1-D21* and showed an HR phenotype with a relatively stunted growth stature. In this background, *Rp1-D21*-suppressed plants due to EMS mutagenesis were easily distinguished from the rest of the M1 siblings because of their non-lesioned and highly robust growth phenotype (Fig. 2A). About 23,000 M1 progenies were screened in field to identify intragenic suppressor mutants that had lost the *Rp1-D21* phenotype. In total, 32 mutants which lacked the HR phenotype of *Rp1-D21* were identified and 14 of them were characterized in detail. Gene specific primers for *Rp1-D21* (S1 Table) were used to sequence the *Rp1-D21* gene in these mutants.

### Plasmid construction

All primers used in this study are listed in S1 Table. In *Rp1-D* and all its paralogs, no intron is found in the open reading frame (ORF) region [20,35], thus we amplified the ORFs of *Rp1-D*, *Rp1-dp2* and *Rp1-D21* from the plasmids gifted by Dr. Scot Hulbert (Washington State University), and cloned them into pENTR directional TOPO cloning vector (D-TOPO, Invitrogen). After sequencing, they were transferred into gateway vectors by LR reactions: pGWB2 (no tag), pGWB14 (with a 3×HA epitope tag in the C-terminus) or pSITEII-N1-EGFP (with EGFP epitopic tag in the C-terminus) [67,68]. *Rp1-D21* was also isolated from maize line *Rp1-D21*-H95 using the primers listed in S1 Table.

The different domains (CC, CC-NB-ARC, NB-ARC, NB-ARC-LRR, and LRR) of Rp1-D21 or Rp1-D were amplified using primer pairs listed in S1 Table. The resulting PCR products were cloned into D-TOPO and sequenced, then transferred into pGWB2, pGWB14 or pSITEII-N1-EGFP by gateway LR reactions.

### Site-directed mutagenesis

Overlapping extension PCR primers (S1 Table) were designed for generating the site-directed mutations: Rp1-D21(K225R), Rp1-D(H517A), Rp1-D(D518V), Rp1-D(H521A), Rp1-D(D522V), Rp1-dp2(D513V), Rp1-dp2(H512A/D513V), Rp1-dp2(H516A), Rp1-dp2(D517V), Rp1-dp2(K225R/D517V) and 12 missense EMS mutations listed in Table 2. The site-directed mutations were cloned into D-TOPO and verified by sequencing and sub-cloned into the gateway vector pGWB14 by LR reaction.

### Sequence alignment and homology structure modeling

NLR sequences were aligned by ClustalW (www.ebi.ac.uk), and edited by BioEdit software. Homology modeling of the NB-ARC domain was performed with Phyre 2 [69] based on the crystal structure of human APAF1 (PDB: 1z6t). The three-dimensional structure and the surface electropotential were mapped using PyMOL (http://www.pymol.org/).

### *Agrobacterium tumefaciens*-mediated transient expression

*Agrobacterium tumefaciens* strain GV3101 (pMP90) transformed with binary vector constructs was grown at 28°C overnight in 5ml L-broth medium supplemented with appropriate antibiotics. The bacteria were collected at 4,000g by centrifugation and resuspended in 2 ml resuspension buffer (10 mM MES pH5.6, 10 mM MgCl_2_ and 200 μM acetosyringone). The final concentration of the bacteria was diluted to the OD_600_ of 0.5 using the same resuspension buffer. To prevent the onset of post-transcriptional gene silencing and improve the efficiency of transient expression, a strain containing p19 protein was included at OD_600_ of 0.2 to all the bacteria strains [70]. The solution was left at room temperature for 3 hrs on bench before infiltration into the abaxial side of *N*. *benthamiana* leaves. After infiltration, plants were put at room temperature with 16h-light/8h-dark. At least 15 individual leaves were infiltrated by different constructs, and each experiment was repeated at least three times.

### Protein analysis

For protein expression analysis, three leaf discs (1.2 cm diameter) from different single plants were collected at 30 hours post infiltration (hpi). The samples were ground with prechilled plastic pestles in liquid nitrogen, and total protein was extracted in 160 μl extraction buffer [20 mM Tris·HCl (pH 8.0), 150 mM NaCl, 1 mM EDTA (pH 8.0), 1% Triton X-100, 0.1% SDS, 10 mM DTT, 40 μM MG132, and 1× plant protein protease inhibitor mixture (Sigma-Aldrich)]. Samples were centrifuged at 14,000 g for 15 min at 4°C, and 12 μl supernatants were mixed with 2× Laemmli buffer and loaded for SDS-PAGE. For Co-IP assay, EGFP- and 3×HA-tagged constructs were transiently co-expressed in *N*. *benthamiana*. Agrobacterium carrying each construct were diluted to a final concentration of OD_600_ = 0.4 plus p19 with OD_600_ = 0.2. Samples were collected at 30 hpi, and proteins were extracted by grinding 0.8 g of leaf tissues in 2.4 ml extraction buffer [50 mM HEPES (pH 7.5), 50 mM NaCl, 10 mM EDTA (pH 8.0), 0.5% Triton 100, 4 mM DTT and 1× plant protein protease inhibitor mixture (Sigma-Aldrich)]. Extracts were centrifuged at 14,000 rpm for 20 min at 4°C, and 2 ml supernatant was mixed with 30 μl anti-GFP microbeads (Miltenyi Biotec) and rotated for 3 hrs at 4°C. The samples were passed through pre-equilibrated MACS separation columns, and washed four times (1 ml, 1 ml, 500 μl and 500 μl) by washing buffer [50 mM HEPES (pH 7.5), 150 mM NaCl, 10 mM EDTA (pH 8.0), 0.2% Triton 100, 4 mM DTT and 1× plant protein protease inhibitor mixture (Sigma-Aldrich)]. The proteins were eluted by 100 μl pre-heated elution buffer and separated by SDS-PAGE. Proteins were transferred to nitrocellulose membrane (Fisher), and analyzed by western blot. HA detection was performed using a 1:350 dilution of anti-HA-HRP (horseradish peroxidase) (Cat# 12013819001, Roche). GFP detection was performed using a 1:8,000 dilution of primary mouse monoclonal anti-GFP (Cat# ab1218, Abcam), followed by hybridization with a 1:15,000 dilution of anti-mouse-HRP second antibody (Cat# A4426, Sigma). The HRP signal was detected by ECL substrate kit (Supersignal west femto chemiluminescent substrate, Thermo Scientific).

## Supporting Information

S1 Fig Transiently expressed of proteins without any tag fusion in *N*. *benthamiana*. (A) Rp1-D21, Rp1-D and Rp1-dp2 proteins without any tag fusion were agro-infiltrated into *N*. *benthamiana*, with an empty vector (EV) as a negative control. A representative leaf was photographed at 3 days post infiltration (dpi). (B) The different domains of Rp1-D21 without any tag fusion were transiently expressed in *N*. *benthamiana*. A representative leaf was photographed at 3 dpi. These experiments were repeated three times with the same results.
(TIF)

S2 Fig The phenotype of the intragenic Rp1-D21 suppressor mutants transiently expressed in *N*. *benthamiana*. (A) HR phenotype of leaves transiently expressing Rp1-D21 and Rp1-D21-derived intragenic suppressor mutations. All proteins indicated were fused with a C-terminal 3×HA tag and were agro-infiltrated into *N*. *benthamiana*. A representative leaf was photographed at 3 days post infiltration (dpi). (B) Detecting the protein expression of the constructs used in (A). Total protein was extracted from agro-infiltrated leaves at 30 hours post infiltration, and anti-HA antibody was used to detect the expression of fused proteins. Equal loading of protein samples was shown by Ponceau-S staining of Rubisco subunit. These experiments were repeated three times with the same results.
(TIF)

S3 Fig Investigating the function of the MHD motifs of Rp1-D. (A) Point mutations in MHD1 and MHD2 motifs from Rp1-D were fused with a C-terminal 3×HA tag and agro-infiltrated into *N*. *benthamiana*. The representative leaves were photographed at 3 days post infiltration. (B) Protein expression analysis of MHD mutations in Rp1-D. Total protein was extracted from agro-infiltrated leaves at 30 hours post infiltration, and anti-HA antibody was used to detect the expression of the fused proteins. Equal loading of protein samples was shown by Ponceau-S staining of Rubisco subunit. Experiments were repeated three times with the same results.
(TIF)

S4 Fig Summary of results from transient expression in *N*. *benthamiana* of a series of C-terminal deletion constructs of CC-NB-ARC_D21_. (A) Schematic diagram of a series of deletion constructs from CC-NB-ARC_D21_ and the derived fragments used for agro-infiltration of *N*. *benthamiana*. The different domains are indicated using different colors: CC (purple); NB-ARC (red). The positions of the amino acids are indicated on the top. HR was scored on a 0 (no HR) to 5 (strong HR) scale. (B) Protein expression analysis of the constructs shown in (A). Total protein was extracted from agro-infiltrated leaves at 30 hours post infiltration, and anti-GFP antibody was used to detect the expression of fused proteins. Equal loading of protein samples was shown by Ponceau-S staining of Rubisco subunit (below right). The experiments were repeated three times with the same results.
(TIF)

S5 Fig Investigating the ability of C-terminal deletions of Rp1-D21 to trigger hypersensitive response (HR) in *N*. *benthamiana*. (A) Schematic diagram of the C-terminal deletions of Rp1-D21 and the derived fragments used for agro-infiltration of *N*. *benthamiana*. The positions of the deletion breakpoints are indicated and their abilities to induce (+) or not induce (-) HR are indicated on the right of each construct. (B) The HR phenotype of the C-terminal deletions of Rp1-D21 observed at 3 dpi (left). All proteins were fused with a C-terminal 3×HA tag and anti-HA antibody was used for detecting the total proteins extracted from the samples indicated at 30 hours post infiltration (right). Equal loading of protein samples was shown by Ponceau-S staining of Rubisco subunit (below right). The experiments were repeated three times with the same results.
(TIF)

S6 Fig Protein expression analysis of constructs from Fig. 6. Chimeric constructs fused with a 3×HA tag were infiltrated into *N*. *benthamiana*. Total protein was extracted from agro-infiltrated leaves at 30 hours post infiltration, and anti-HA antibody was used to detect the expression of the fused proteins. Equal loading of protein samples was shown by Ponceau-S staining of Rubisco subunit. The experiments were performed three times with similar results.
(TIF)

S7 Fig Intra-molecular interactions from different Rp1 proteins. (A) Inter-domain interactions between the CC and NB-ARC domains in different Rp1 proteins. EGFP- and 3×HA-tagged proteins were transiently co-expressed in *N*. *benthamiana* and samples were collected at 30 hours post infiltration for Co-IP assays. Protein extracts were immunoprecipitated by anti-GFP microbeads and detected by anti-GFP and anti-HA antibodies. (B) Inter-domain interactions between NB-ARC and LRR in different Rp1 proteins. (C) Inter-domain interactions between NB-ARC_D21_ and LRR_V1_, LRR_V1(K1184N)_ or LRR_V18_. These experiments were performed three times with similar results.
(TIF)

S8 Fig Investigating the function of the intragenic suppressor mutations in CC_D21_. (A) Self-association of different CC domain variants. EGFP- and 3×HA-tagged proteins were transiently co-expressed in *N*. *benthamiana* and samples were collected at 30 hours post infiltration for Co-IP assay. Protein extracts were immunoprecipitated by anti-GFP microbeads and detected by anti-GFP and anti-HA antibodies. (B) Investigating the inter-domain interactions between NB-ARC_D21_ and the CC variants. These experiments were performed three times with similar results.
(TIF)

S1 Table Primer sequences used in this study. (DOC)
