# Correction: Molecular and Functional Analyses of a Maize Autoactive NB-LRR Protein Identify Precise Structural Requirements for Activity

**DOI:** 10.1371/journal.ppat.1004830

**Published:** 2015-04-10

**Authors:** Guan-Feng Wang, Jiabing Ji, Farid EI-Kasmi, Jeffery L. Dangl, Guri Johal, Peter J. Balint-Kurti

The authors would like to correct an error in the Author Contribution section. Jeffery L. Dangl (JLD) should be listed as one of the persons who wrote the paper.

The correct author contributions are:

Conceived and designed the experiments: GFW JJ JLD GJ PJBK. Performed the experiments: GFW JJ FEK. Analyzed the data: GFW JJ FEK GJ PJBK. Contributed reagents/materials/analysis tools: GFW PJBK. Wrote the paper: GFW FEK JLD PJBK.
