# Supplementary figures and images for: Molecular and Functional Analyses of a Maize Autoactive NB-LRR Protein Identify Precise Structural Requirements for Activity
Source: PLoS Pathog. 2015 Feb 26;11(2):e1004674. doi: 10.1371/journal.ppat.1004674 (PMC4342346; doi:10.1371/journal.ppat.1004674)

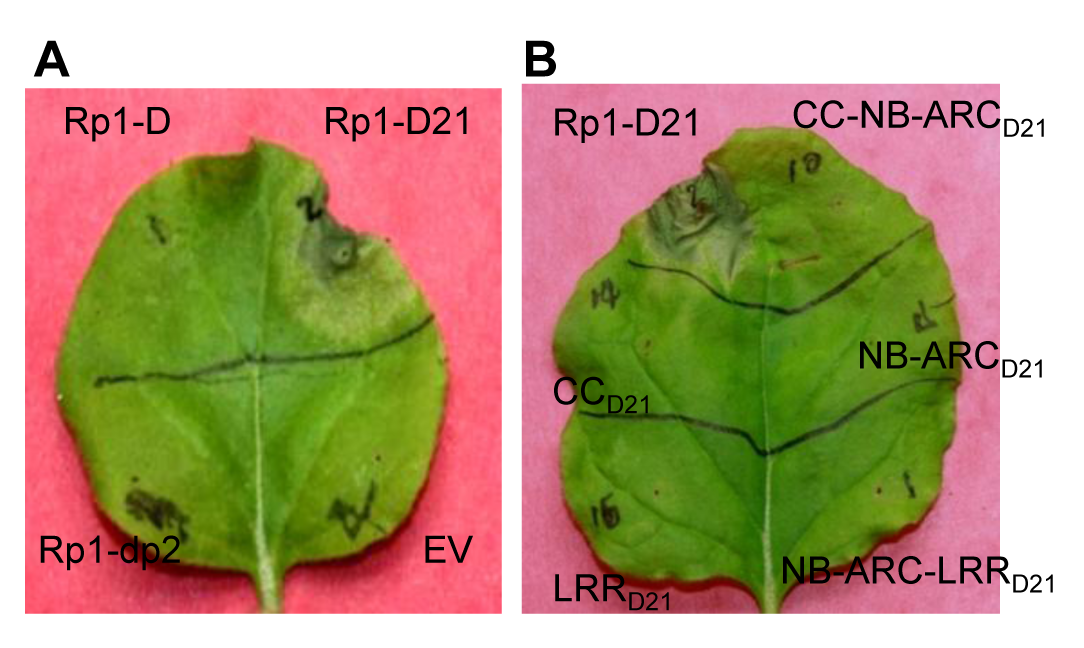

Supplement: S1 Fig — (A) Rp1-D21, Rp1-D and Rp1-dp2 proteins without any tag fusion were agro-infiltrated into N. benthamiana, with an empty vector (EV) as a negative control. A representative leaf was photographed at 3 days post infiltration (dpi). (B) The different domains of Rp1-D21 without any tag fusion were transiently expressed in N. benthamiana. A representative leaf was photographed at 3 dpi. These experiments were repeated three times with the same results. (TIF) [file ppat.1004674.s001.tif]

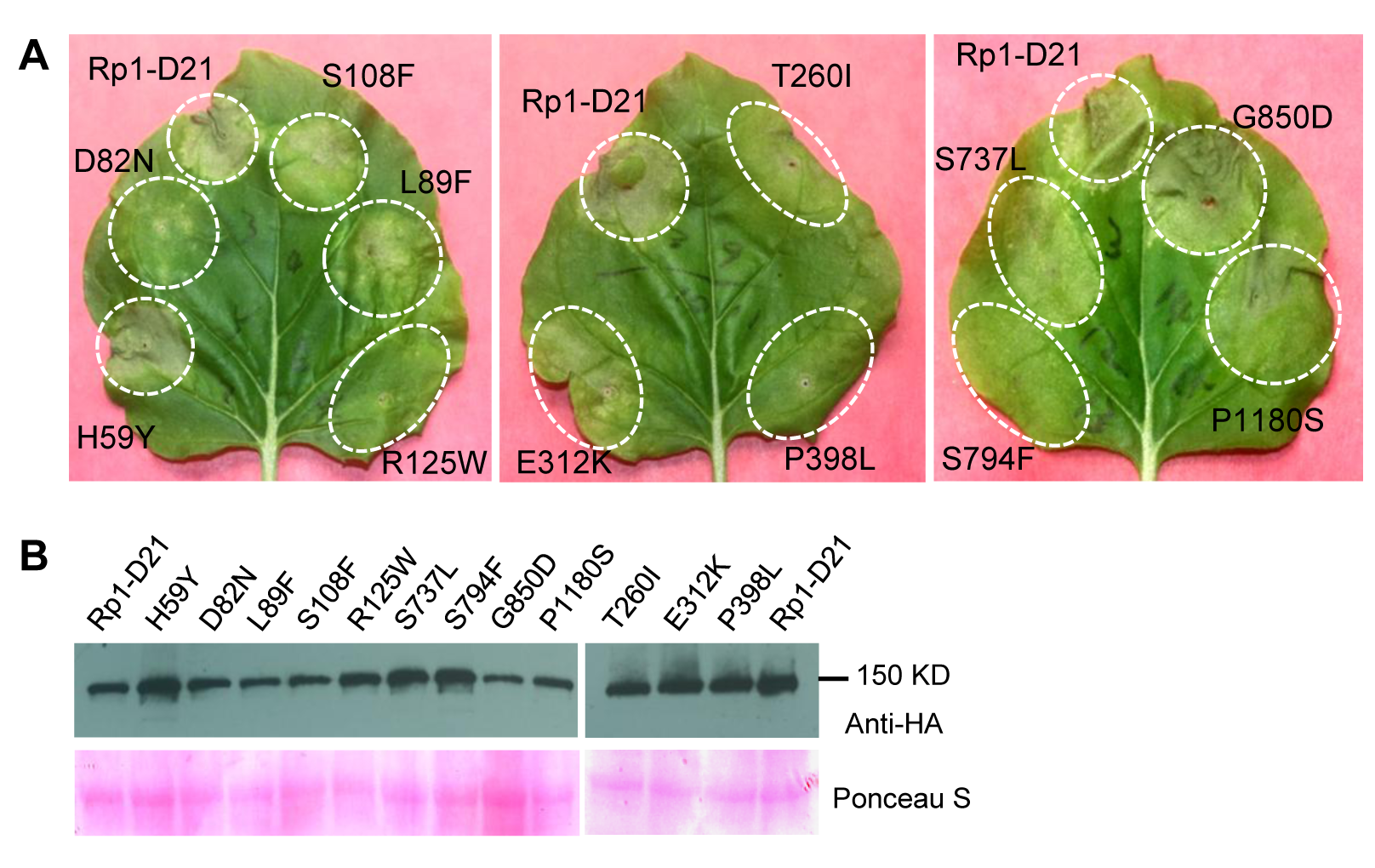

Supplement: S2 Fig — (A) HR phenotype of leaves transiently expressing Rp1-D21 and Rp1-D21-derived intragenic suppressor mutations. All proteins indicated were fused with a C-terminal 3×HA tag and were agro-infiltrated into N. benthamiana. A representative leaf was photographed at 3 days post infiltration (dpi). (B) Detecting the protein expression of the constructs used in (A). Total protein was extracted from agro-infiltrated leaves at 30 hours post infiltration, and anti-HA antibody was used to detect the expression of fused proteins. Equal loading of protein samples was shown by Ponceau-S staining of Rubisco subunit. These experiments were repeated three times with the same results. (TIF) [file ppat.1004674.s002.tif]

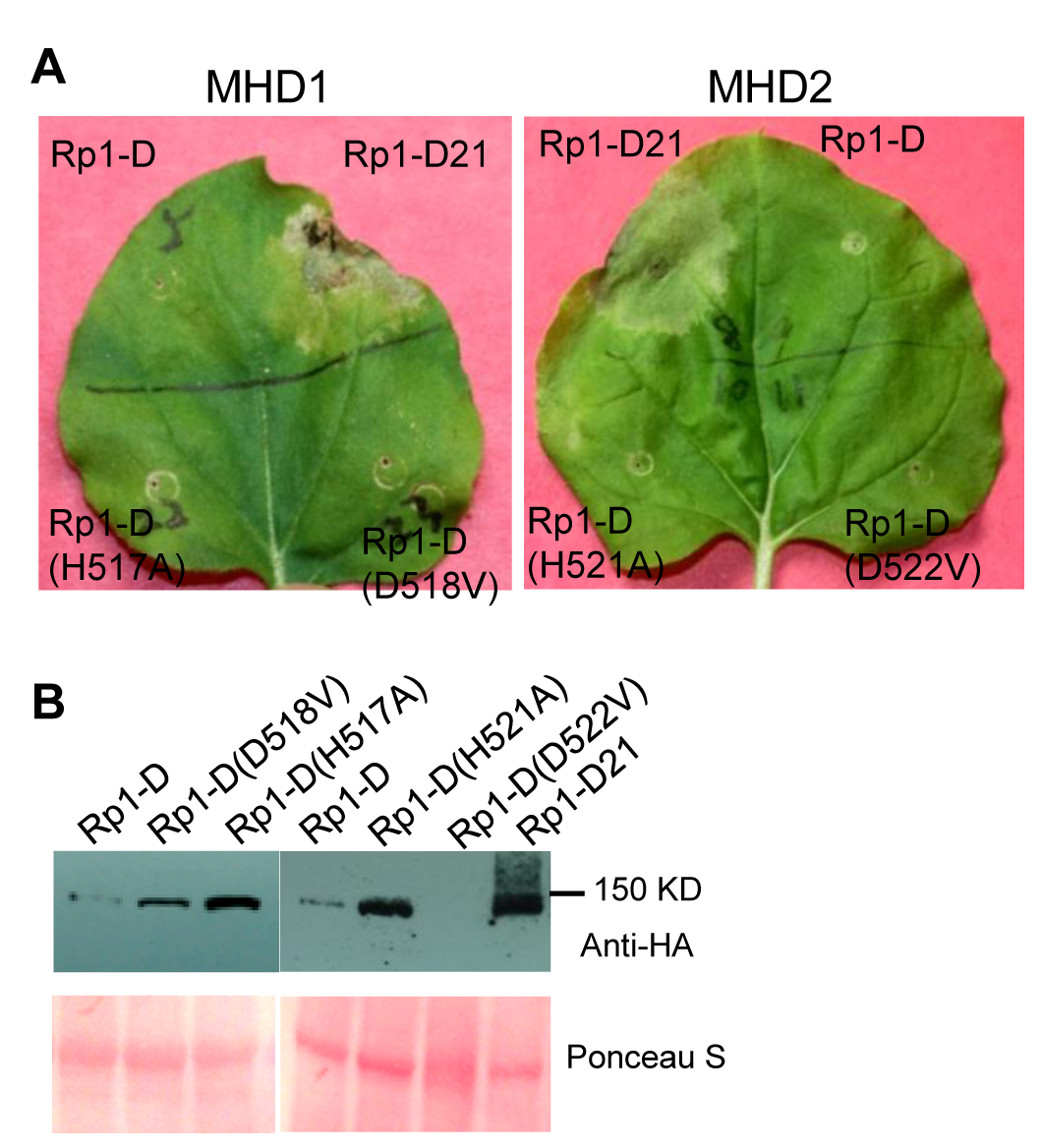

Supplement: S3 Fig — (A) Point mutations in MHD1 and MHD2 motifs from Rp1-D were fused with a C-terminal 3×HA tag and agro-infiltrated into N. benthamiana. The representative leaves were photographed at 3 days post infiltration. (B) Protein expression analysis of MHD mutations in Rp1-D. Total protein was extracted from agro-infiltrated leaves at 30 hours post infiltration, and anti-HA antibody was used to detect the expression of the fused proteins. Equal loading of protein samples was shown by Ponceau-S staining of Rubisco subunit. Experiments were repeated three times with the same results. (TIF) [file ppat.1004674.s003.tif]

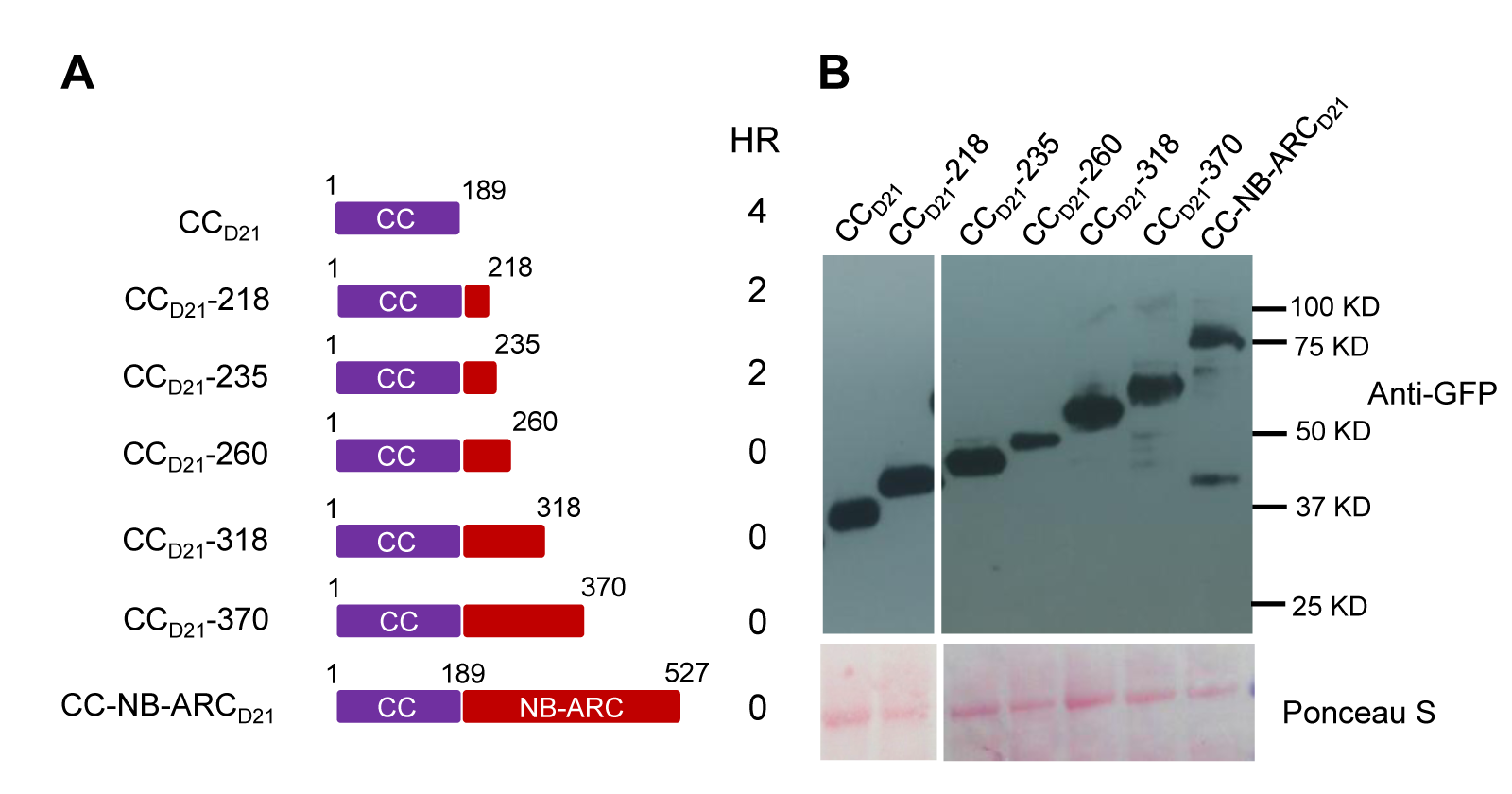

Supplement: S4 Fig — (A) Schematic diagram of a series of deletion constructs from CC-NB-ARCD21 and the derived fragments used for agro-infiltration of N. benthamiana. The different domains are indicated using different colors: CC (purple); NB-ARC (red). The positions of the amino acids are indicated on the top. HR was scored on a 0 (no HR) to 5 (strong HR) scale. (B) Protein expression analysis of the constructs shown in (A). Total protein was extracted from agro-infiltrated leaves at 30 hours post infiltration, and anti-GFP antibody was used to detect the expression of fused proteins. Equal loading of protein samples was shown by Ponceau-S staining of Rubisco subunit (below right). The experiments were repeated three times with the same results. (TIF) [file ppat.1004674.s004.tif]

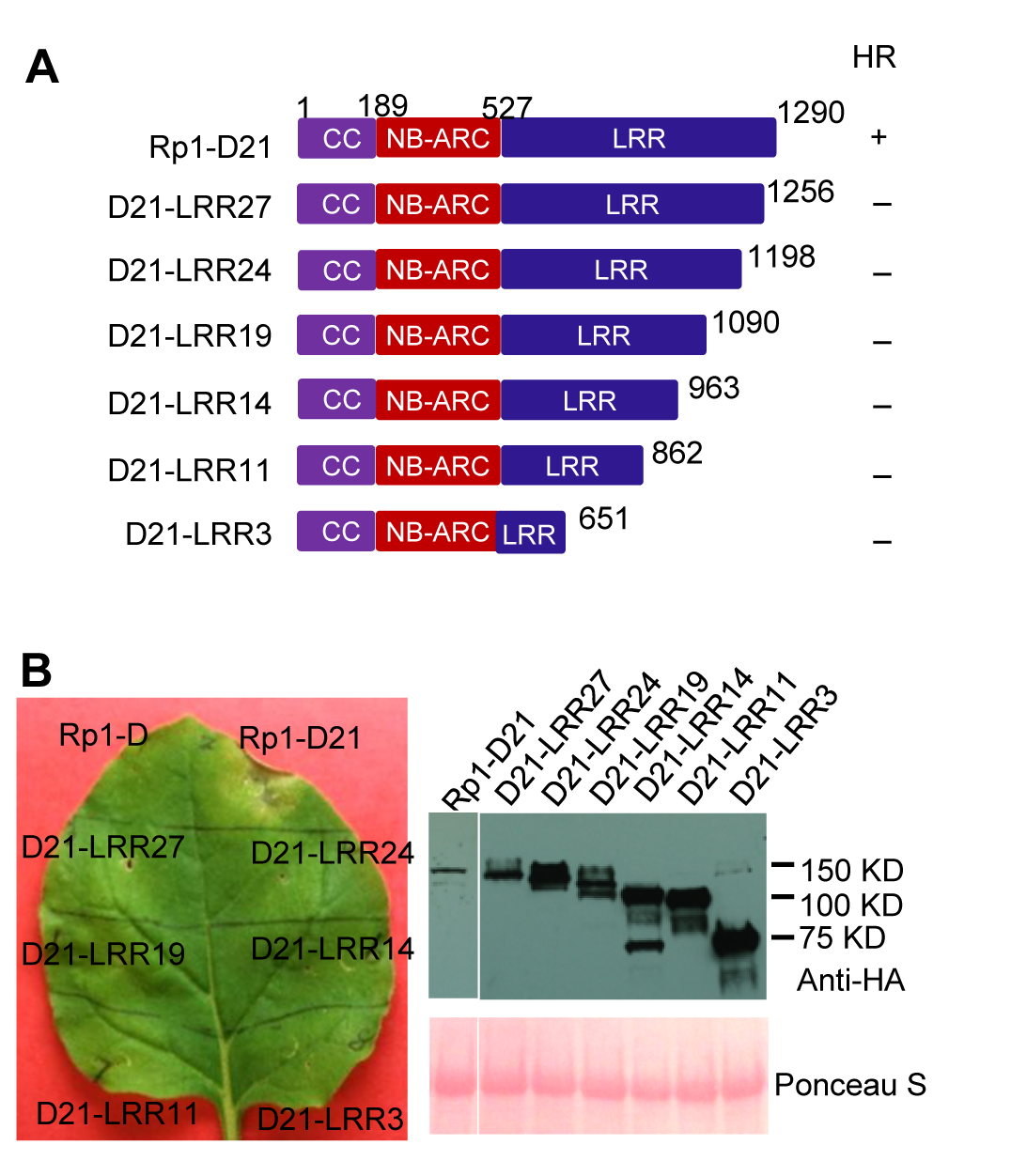

Supplement: S5 Fig — (A) Schematic diagram of the C-terminal deletions of Rp1-D21 and the derived fragments used for agro-infiltration of N. benthamiana. The positions of the deletion breakpoints are indicated and their abilities to induce (+) or not induce (-) HR are indicated on the right of each construct. (B) The HR phenotype of the C-terminal deletions of Rp1-D21 observed at 3 dpi (left). All proteins were fused with a C-terminal 3×HA tag and anti-HA antibody was used for detecting the total proteins extracted from the samples indicated at 30 hours post infiltration (right). Equal loading of protein samples was shown by Ponceau-S staining of Rubisco subunit (below right). The experiments were repeated three times with the same results. (TIF) [file ppat.1004674.s005.tif]

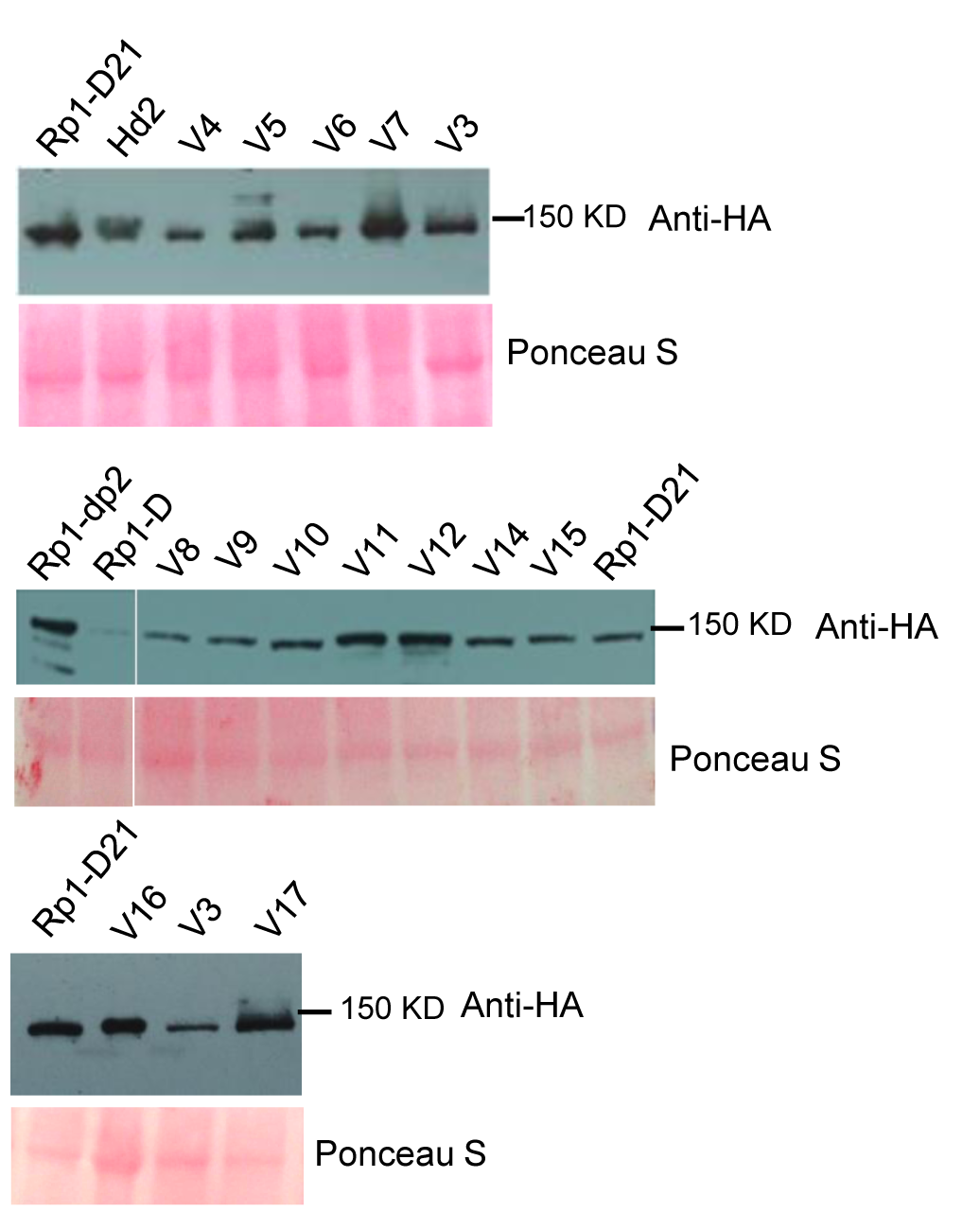

Supplement: S6 Fig — Chimeric constructs fused with a 3×HA tag were infiltrated into N. benthamiana. Total protein was extracted from agro-infiltrated leaves at 30 hours post infiltration, and anti-HA antibody was used to detect the expression of the fused proteins. Equal loading of protein samples was shown by Ponceau-S staining of Rubisco subunit. The experiments were performed three times with similar results. (TIF) [file ppat.1004674.s006.tif]

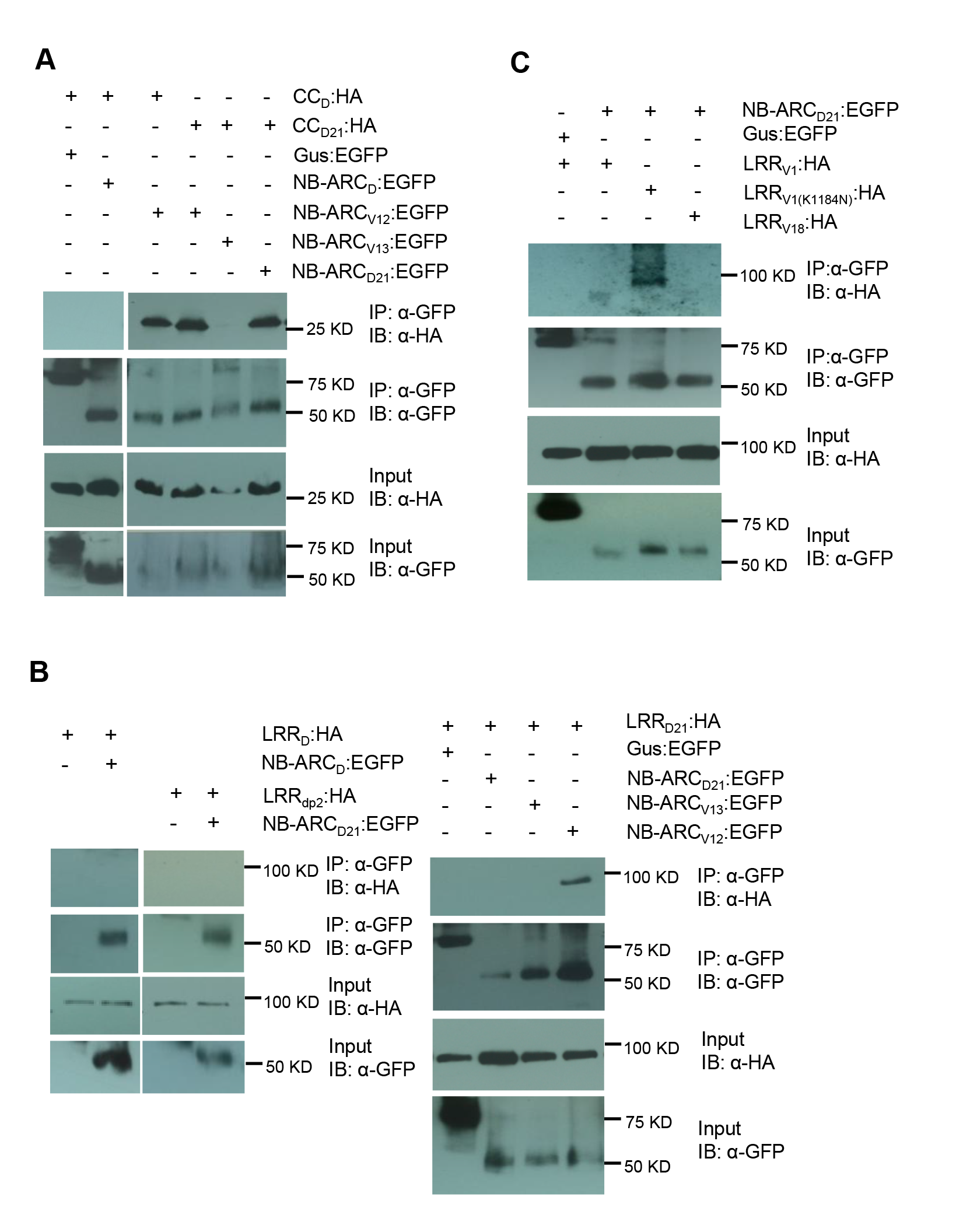

Supplement: S7 Fig — (A) Inter-domain interactions between the CC and NB-ARC domains in different Rp1 proteins. EGFP- and 3×HA-tagged proteins were transiently co-expressed in N. benthamiana and samples were collected at 30 hours post infiltration for Co-IP assays. Protein extracts were immunoprecipitated by anti-GFP microbeads and detected by anti-GFP and anti-HA antibodies. (B) Inter-domain interactions between NB-ARC and LRR in different Rp1 proteins. (C) Inter-domain interactions between NB-ARCD21 and LRRV1, LRRV1(K1184N) or LRRV18. These experiments were performed three times with similar results. (TIF) [file ppat.1004674.s007.tif]

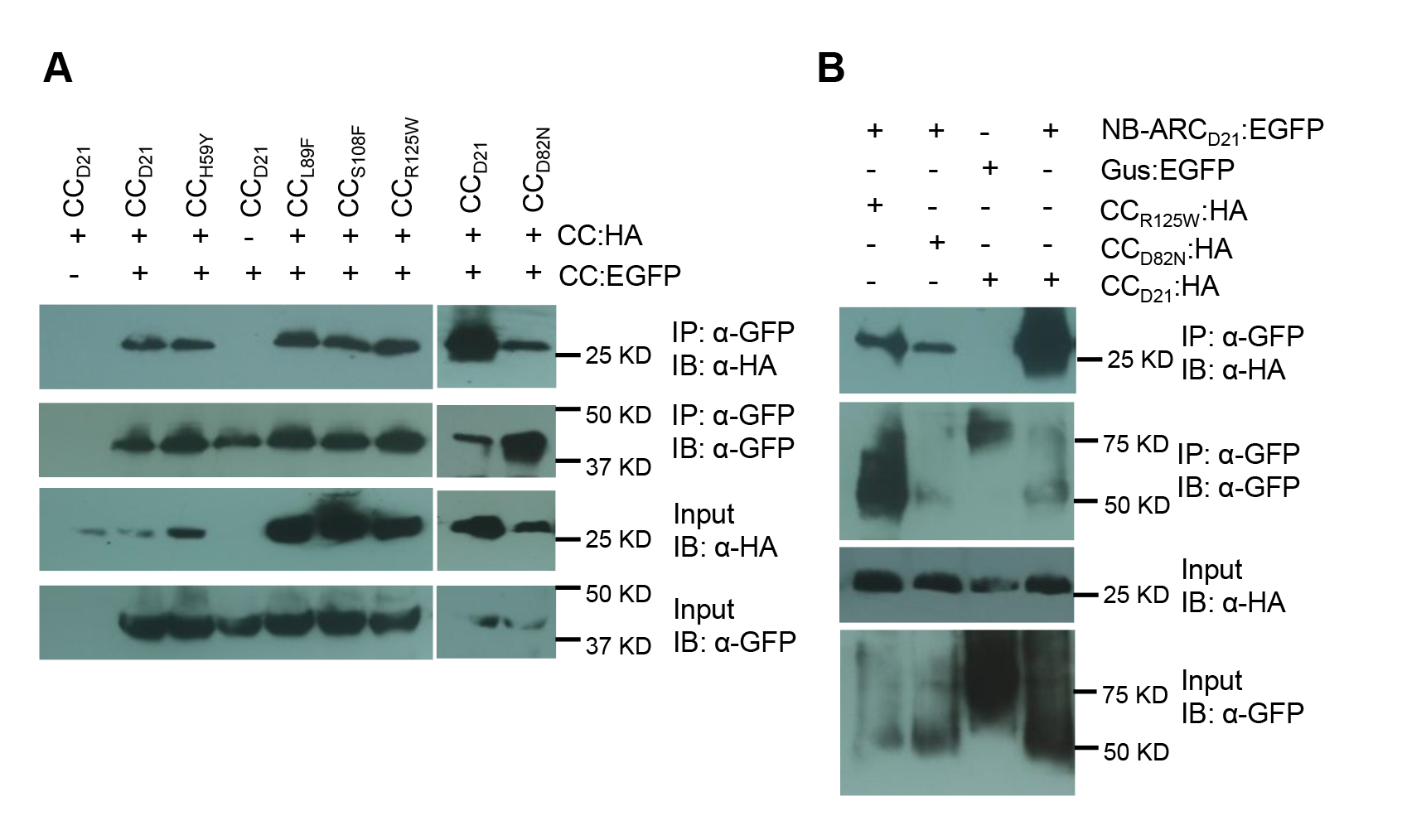

Supplement: S8 Fig — (A) Self-association of different CC domain variants. EGFP- and 3×HA-tagged proteins were transiently co-expressed in N. benthamiana and samples were collected at 30 hours post infiltration for Co-IP assay. Protein extracts were immunoprecipitated by anti-GFP microbeads and detected by anti-GFP and anti-HA antibodies. (B) Investigating the inter-domain interactions between NB-ARCD21 and the CC variants. These experiments were performed three times with similar results. (TIF) [file ppat.1004674.s008.tif]
