# Supplementary material for: Molecular and Functional Analyses of a Maize Autoactive NB-LRR Protein Identify Precise Structural Requirements for Activity
Source: PLoS Pathog. 2015 Feb 26;11(2):e1004674. doi: 10.1371/journal.ppat.1004674 (PMC4342346; doi:10.1371/journal.ppat.1004674)
Supplement: S1 Table — (DOC) [file ppat.1004674.s009.doc]

**S1Table.** Primer sequences used in this study.

| | Primer name | Sequence(5'-3') | Usage | | --- | --- | --- | |
| --- | --- | --- | --- |
| | Rp1-GF1 | CACCATGGCCGACTTGGCGCTCG | Amplifying Rp1-D full-length sequence and  cloning it into entry vector pENTR/TOPO | | --- | --- | --- | | Rp1D-GR1 | AGGAAGCCAGATCGATTTTGGTGGAAAGC | | Rp1D21-GF1 | CACCATGGCCGACTTCGCGCTCG | Amplifying Rp1-D21 full-length sequence and  cloning it into entry vector pENTR/TOPO | | Rp1D-GR1 | AGGAAGCCAGATCGATTTTGGTGGAAAGC | | Rp1D21-GF1 | CACCATGGCCGACTTCGCGCTCG | Amplifying Rp1-dp2 full-length sequence and  cloning it into entry vector pENTR/TOPO | | Rp1-dp2-R1 | AAAAAAAAAGATCGATATTGGTAGAAAGCTC | | Rp1D21-GF1 | CACCATGGCCGACTTCGCGCTCG | Amplifying the CC domain of Rp1-D21 | | Rp1-CC-R1 | CCTGTCGCGACCAAAAACCTTGG | | Rp1-NB-F1 | CACCATGGATCGTGATCGTATAGTAG | Amplifying the NB domain of Rp1-D21 | | Rp1-ARC-R1 | GCAGTCTTCTCTAGAGAGTGACTCTG | | Rp1D-LRR-F3 | CACCATGGACTGCTTTAGATTAGAAGATG | Amplifying the LRR domain of Rp1-D21 | | Rp1D-GR1 | AGGAAGCCAGATCGATTTTGGTGGAAAGC | | Rp1D21-GF1 | CACCATGGCCGACTTCGCGCTCG | Amplifying the CC-NB domain of Rp1-D21 | | Rp1-ARC-R1 | GCAGTCTTCTCTAGAGAGTGACTCTG | | Rp1-NB-F1 | CACCATGGATCGTGATCGTATAGTAG | Amplifying the NB-LRR domain of Rp1-D21 | | Rp1D-GR1 | AGGAAGCCAGATCGATTTTGGTGGAAAGC | | Rp1D21-218R | AACAATGGCCAAACCCGAGTAC | Combining with Rp1D21-GF1 to amplify CC_D21_-218 | | Rp1D21-235R | GTCATTATAGACATACTGTGCTAAGGTGG | Combining with Rp1D21-GF1 to amplify CC_D21_-235 | | Rp1D21-260R | TGTGTGACGATGCACATCAAGTTTGCG | Combining with Rp1D21-GF1 to amplify CC_D21_-260 | | Rp1D21-318R | AGCAAGGAATAACTCCCACTCTGTCTC | Combining with Rp1D21-GF1 to amplify CC_D21_-318 | | Rp1D21-370R | GATTTCTGCTCCAGAGAAAGCATGGTG | Combining with Rp1D21-GF1 to amplify CC_D21_-370 | | LRR-1256R | GCAATTCAATATAGTTATGCGCTGGAG | Combining with Rp1D21-GF1 to amplify D21-LRR27 | | LRR-1198R | TTCTTCAAATGAAACTGACGGCTCC | Combining with Rp1D21-GF1 to amplify D21-LRR24 | | LRR-1090R | ATGTGGCAAGCCATTAATGAACGAATC | Combining with Rp1D21-GF1 to amplify D21-LRR19 | | LRR-963R | TGGCATCTCCATGGCCCTTCC | Combining with Rp1D21-GF1 to amplify D21-LRR14 | | LRR-862R | CTCATTGTTGGTGATAAACATAAGCAG | Combining with Rp1D21-GF1 to amplify D21-LRR11 | | LRR-651R | AGGCAAATTCTCCACCATGTGGTTTAACC | Combining with Rp1D21-GF1 to amplify D21-LRR3 | | H59Y-F1 | CAGAAGAGCCCCTACAGGGGCATACTGGAG | Overlapping primers used for generate site-directed mutant H59Y | | H59Y-R1 | CTCCAGTATGCCCCTGTAGGGGCTCTTCTG | | D82N-F1 | GAGGACTTGTTGAACGAGCATGAGTAC | Overlapping primers used for generate site-directed mutant D82N | | D82N-R1 | GTACTCATGCTCGTTCAACAAGTCCTC | | L89F-F1 | CATGAGTACAATGTCtTTGAAGGCAAGGCCAAG | Overlapping primers used for generate site-directed mutant L89F | | L89F-R1 | CTTGGCCTTGCCTTCAAAGACATTGTACTCATG | | S108F-F1 | GAGCATGGAAGCTCCTTCACTGCAACTACTG | Overlapping primers used for generate site-directed mutant S108F | | S108F-R1 | CAGTAGTTGCAGTGAAGGAGCTTCCATGCTC | | R125W-F1 | CATGAGCAGGGCGTGGAACTTGCTCCCTC | Overlapping primers used for generate site-directed mutant R125W | | R125W-R1 | GAGGGAGCAAGTTCCACGCCCTGCTCATG | | T260I-F1 | GATGTGCATCGTCACATAAGGGAGATTATGGAG | Overlapping primers used for generate site-directed mutant T260I | | T260I-R1 | CTCCATAATCTCCCTTATGTGACGATGCACATC | | E312K-F1 | CTCATAATGAGACAAAGTGGGAGTTATTC | Overlapping primers used for generate site-directed mutant E312K | | E312K-R1 | GAATAACTCCCACTTTGTCTCATTATGAG | | P398L-F1 | CTTGGACAATGTCTTTTGGCAGC | Overlapping primers used for generate site-directed mutant P398L | | P398L-R1 | GCTGCCAAAAGACATTGTCCAAG | | S737L-F1 | GATGAAGCCGTAGAGTTGAAGCTATATCTG | Overlapping primers used for generate site-directed mutant S737L | | S737L-R1 | CAGATATAGCTTCAACTCTACGGCTTCATC | | S794F-F1 | GGTTACTAGAGCGATTCTATTTTGAGAATTTG | Overlapping primers used for generate site-directed mutant S794F | | S794F-R1 | CAAATTCTCAAAATAGAATCGCTCTAGTAACC | | G850D-F1 | CAGATTTATCAATTGATTGGTGCCCACTGC | Overlapping primers used for generate site-directed mutant G850D | | G850D-R1 | GCAGTGGGCACCAATCAATTGATAAATCTG | | P1180S-F1 | GAAGGGTTTACAGCCTCACCAAATCTTACTC | Overlapping primers used for generate site-directed mutant P1180S | | P1180S-R1 | GAGTAAGATTTGGTGAGGCTGTAAACCCTTC | | K225R-F1 | GGAATGGGGAGGTCCACCTTAGCACAG | Overlapping primers used for generate site-directed  mutant Rp1-D21(K225R) | | K225R-R1 | CTGTGCTAAGGTGGACCTCCCCATTCC | | Rp1-D-MF1 | ATGCATGTTATCCTTCATGATTTTGC | Overlapping primers used for generate site-directed  mutants Rp1-D(D518V) and Rp1-dp2(D513V) | | Rp1-D-MR1 | ATGAAGGATAACATGCATGACATAGTAC | | Rp1-D-MF2 | ATGGCTGATATCCTTCATGATTTTGC | Overlapping primers used for generate site-directed  mutants Rp1-D(H517A) | | Rp1-D-MR2 | ATGAAGGATATCAGCCATGACATAGTAC | | Rp1-D-MF3 | ATGGCTGTTATCCTTCATGATTTTGC | Overlapping primers used for generate site-directed  mutants Rp1-dp2(H512A/D513V) | | Rp1-D-MR3 | ATGAAGGATAACAGCCATGACATAGTAC | | Rp1D-H521A-F1 | TATCCTTGCTGATTTTGCAGAGTC | Overlapping primers used for generate site-directed  mutants Rp1D(H521A) and Rp1-dp2(H516A) | | Rp1D-H521A-R1 | GACTCTGCAAAATCAGCAAGGATA | | Rp1D-D522V-F1 | TATCCTTCATGTCTTTGCAGAGTC | Overlapping primers used for generate site-directed  mutants Rp1D(D522V) and Rp1-dp2(D517V) | | Rp1D-D522V-R1 | GACTCTGCAAAGACATGAAGGATA | |
